# Meta-regression of randomized control trials with antithrombotics: weak correlation between net clinical benefit and all cause-mortality

**DOI:** 10.1038/s41598-021-94160-1

**Published:** 2021-07-19

**Authors:** Roubi Kilo, Silvy Laporte, Rama Arab, Sabine Mainbourg, Steeve Provencher, Guillaume Grenet, Laurent Bertoletti, Laurent Villeneuve, Michel Cucherat, Jean-Christophe Lega, META-EMBOL Group

**Affiliations:** 1 Hospices Civils de Lyon, Hôpital Lyon Sud, Service de Recherche Et D’Epidémiologie Cliniques, Lyon, France; 2 Unité De Recherche Clinique, Innovation, Pharmacologie, Hôpital Nord, CHU De Saint-Étienne, Saint-Etienne, France; 3 INSERM, UMR1059, Equipe Dysfonction Vasculaire Et Hémostase, Université Jean-Monnet, Saint-Etienne, France; 4 Service De Médecine Interne Et Vasculaire, Hospices Civils De Lyon, Hôpital Lyon Sud, Lyon, France; 5 Univ Lyon, Université Claude Bernard Lyon 1 - UMR CNRS 5558, Laboratoire de Biométrie Evolutive, Equipe Evaluation Et Modélisation Des Effets Thérapeutiques, Lyon, France; 6 Pneumologue, Centre De Recherche De L’institut Universitaire De Cardiologie Et De Pneumologie De Québec, Québec, Canada; 7 Service Hospitalo Universitaire de PharmacoToxicologie, Pôle de Santé Publique, Hopsices Civils De Lyon, Lyon, France; 8 Service De Médecine Vasculaire Et Thérapeutique, Chu de Saint-Étienne, France; 9 INSERM, UMR1059, Equipe Dysfonction Vasculaire Et Hémostase, Université Jean-Monnet; INSERM, CIC-1408, CHU Saint-Etienne, 42055 Saint-Etienne, France; 10 Hospices Civils de Lyon, Hôpital Lyon Sud, Service de Recherche Et D’Epidémiologie Cliniques, 69495 Pierre-Bénite, France; 11 Université Lyon-1, EA 3738 CICLY, 69921 Oullins Cedex, Lyon, France; 12 Pôle de Santé Publique, Hospices Civils de Lyon, Hôpital Lyon Sud, Chemin du Grand Revoyet, 69495 Pierre-Bénite, France

**Keywords:** Drug safety, Pharmaceutics, Pharmacology, Drug discovery, Evolution, Biomarkers, Cardiology, Diseases, Medical research, Molecular medicine

## Abstract

This study aimed to explore the validity of the use of the net clinical benefit (NCB), i.e. the sum of major bleeding and thrombotic events, as a potential surrogate for all-cause mortality in clinical trials assessing antithrombotics. Published randomized controlled trials testing anticoagulants in the prevention or treatment of venous thromboembolism (VTE) and non-valvular atrial fibrillation (NVAF) were systematically reviewed. The validity of NCB as a surrogate endpoint was estimated by calculating the strength of correlation of determination (R^2^) and its 95% confidence interval (CI) between the relative risks of NCB and all-cause mortality. Amongst the 125 trials retrieved, the highest R^2^_trial_ values were estimated for NVAF (R^2^_trial_ = 0.41, 95% CI [0.03; 0.48]), and acute VTE (R^2^_trial_ = 0.30, 95% CI [0.04; 0.84]). Conversely, the NCB did not correlate with all-cause mortality in prevention studies with medical (R^2^_trial_ = 0.12, 95% CI [0.00; 0.36]), surgical (R^2^_trial_ = 0.05, 95% CI [0.00; 0.23]), and cancer patients (R^2^_trial_ = 0.006, 95% CI [0.00; 1.00]). A weak correlation between NCB and all cause-mortality was found in NVAF and acute VTE, whereas no correlation was observed in clinical situations where the mortality rate was low. Consequently, NCB should not be considered a surrogate outcome for all cause-mortality in anticoagulation trials.

## Introduction

Non-valvular atrial fibrillation (NVAF) and venous thromboembolism (VTE), which includes deep vein thrombosis (DVT) and pulmonary embolism (PE), are two common diseases associated with significant morbidity and mortality^1^. Furthermore, patients hospitalized for medical illness, undergoing surgery, and cancer patients have a higher risk of venous thromboembolism or bleeding events than the general population^2–4^. As they reduce the risk of death and injury related to thrombotic events, anticoagulants are the cornerstone of the management of these cardiovascular diseases^5^.

Since their discovery, the use of vitamin K antagonists (VKA) has been shown to reduce the mortality related to cardiovascular diseases^6^. However, due to their narrow therapeutic index, bleeding events are the most important complications related to the use of VKA, and the incidence of major bleeding ranges from 1.4 to 3.4% in NVAF patients^7,8^ and from 0 to 4.3% in VTE patients^9^. More recently, direct oral anticoagulants (DOAC) have been shown to be non-inferior to VKA in preventing thrombotic events while being associated with a reduction of major bleedings, and thus became the standard therapy and preventive treatment for VTE and NVAF^10,11^.

The positive impact of anticoagulants on cardiovascular mortality is presumably a result of the reduced amount of thrombotic events. However, major bleeding is also associated with significant morbidity and mortality^12,13^. To take into account the balance between potential benefits (i.e. reduced risk of thromboembolism) and harm (i.e. increased risk of major bleeding) in randomized trials, the concept of net clinical benefit (NCB), which is the sum of major bleeding and thrombotic events, appeared recently as a potentially relevant outcome in phase III clinical trials evaluating antithrombotics^14^. These measurable events lie within the pathophysiological spectrum of NVAF and VTE, allowing the summarization of treatment effects and increasing the number of total events, thus increasing the study power. Technically, to evaluate the value of NCB as a surrogate endpoint in clinical trials, a linear correlation between the treatment effects on the surrogate and on the final outcome needs to be established for each indication separately, and its strength has to be checked in advance with several statistics analytic methods^15^. However, direct evidence of the association between the treatment effects of anticoagulation on the NCB and cardiovascular mortality in the setting of clinical trials is lacking.

The present study aimed to explore the validity of the NCB as a potential surrogate for all-cause mortality in trials testing antithrombotics for the prevention and treatment of VTE and NVAF.

## Materials and methods

The present study was conducted according to the PRISMA statement^16^.

### Search strategy and study identification

First, all published randomized controlled trials registered in the META-EMBOL database (Silvy Laporte, University of Saint-Etienne, PHRC 2008) were investigated. This database collected the results of trials assessing the efficacy of antithrombotics in the prevention or treatment of VTE and NVAF^17^.

Additional studies were searched for on electronic databases such as MEDLINE, the Cochrane Library databases, and EMBASE from 1990 to December 2020, in English and non-English language by using sensitive keywords to detect all the studies (see online supplement).

Hand searching through medical journals, reviews, and bibliography of each selected article was carried out to identify additional studies that were not reported in those electronic databases and META-EMBOL.

### Study selection

The database was screened by two authors (R.K and R.A) independently to identify studies that potentially met the eligibility criteria. These were: randomized controlled trials, parallel groups, open or double-blind design evaluating antithrombotics compared to placebo or control treatment for (1) VTE, (2) thromboprophylaxis in hospitalized patients for medical conditions, major orthopedic, and/or abdominal surgery, (3) thromboprophylaxis in cancer patients, and/or (4) NVAF. Also, for inclusion studies needed to report the three outcomes of interest: thrombosis, major bleeding, and all-cause mortality. Disagreements about inclusion were resolved by consensus or by consulting a third author (J.-C.L.).

### Definition of outcomes

The net clinical benefit (NCB) was computed in each arm by adding major bleeding events and thrombosis events retrospectively in each study. The major bleeding in non‐surgical patients was defined according to the International Society on Thrombosis and Haemostasis (ISTH) criteria^18^, i.e. fatal bleeding, bleeding manifest in a critical organ such as intracranial bleeding, and/or explicit bleeding correlated with a decrease of hemoglobin level of 20 g/L or more, or necessitating a transfusion of at least two units of red cells or whole blood. Major bleeding for surgical patients was defined according to the European Agency for the Evaluation of Medical Products definition, i.e. bleeding detected at the surgical site and conducting to re-operation or any special medical intervention, in addition to the criteria mentioned above.

The effects of the assessed treatments on thrombotic events were evaluated from the main pre-specified efficacy outcome of each trial. All-cause mortality was used irrespective of its relationship with the cardiovascular event.

### Data extraction

When a trial met the eligibility criteria, two authors separately extracted the following data in addition to thrombotic and bleeding events: name of the first author and year(s) of publication, study acronym, study design, disease, treatment regimens, class of comparison, and study size.

### Statistical analysis

For each condition (acute treatment of VTE, treatment of NVAF, and VTE prevention in medical, surgical, and cancer patients), a meta-analysis was conducted and forest plots were generated and computed using the random-effects model to estimate the relative risk (RR) for all-cause mortality (standard outcome) and NCB (surrogate outcome), as well as the corresponding 95% confidence intervals (CI) in patients treated (in the experimental anticoagulation group) compared to patients in control groups. Additional meta-analysis and forest plots were generated for each class of medicine separately (Antiplatelet, VKA, DOAC, and LMWH) and calculated the RR for all-cause mortality and NCB. Adjusted continuity corrections of 0.5 were used for any study with no event^19^.

To validate NCB as a surrogate of all-cause mortality, the method reported by Buyse et al.^20^ was used. A linear regression model was therefore used to assess the association between the RR for NCB and the RR for all-cause mortality by calculating the coefficient of determination (R^2^_trial_). The percentile bootstrapping method (resampling 1000 times) was applied in order to obtain a high accuracy to compute the 95% confidence interval for R^2^_trial_ in addition to the prediction interval. The validity of the surrogate endpoints depends on the strength of the association with the ultimate endpoint. Indeed, the coefficient of determination R^2^_trial_ should be more than 0.65 and close to 1^21^. In practice, an R^2^_trial_ with an upper limit of the 95% confidence interval (95% CI) ≤ 0.7 (i.e. limited correlation) confirms the lack of validity of the surrogate endpoint, whereas an R^2^_trial_ with a lower limit of the 95% confidence interval ≥ 0.85 supports the validity of the surrogate. In case of intermediate correlation (0.7 < R_trial_ < 0.85), the validation of the surrogate endpoints remains unclear^15^.

The surrogate threshold effect (STE) can also be assessed to estimate the minimal treatment effect on the surrogate endpoint predicting a significantly nonzero effect on the true endpoint^22,23^. To compute the STE, the linear regression model was calculated and the 95% prediction intervals were plotted. The value of the STE is the value on the x-axis (log RR of NCB) for which the lower limit of the prediction interval meets a point corresponding to 1 (zero effect on the true endpoint) on the y-axis (log RR of all-cause mortality).

Furthermore, two sensitivity analyses were performed to assess the robustness of the results. The first analysis included only studies with a double-blind design, whereas the second one was applied to studies that included only the new direct oral anticoagulants (except ximelagatran) in the experimental arm.

The association between the mortality rate and the relative risk reduction was explored using linear regression for all indications together.

The linear regression models were performed using the statistical software R, version 3.5.2^24^ with the META, METAFOR, and GGPLOT^25^ packages.

## Results

### Search results and characteristics of studies

**Figure 1:**
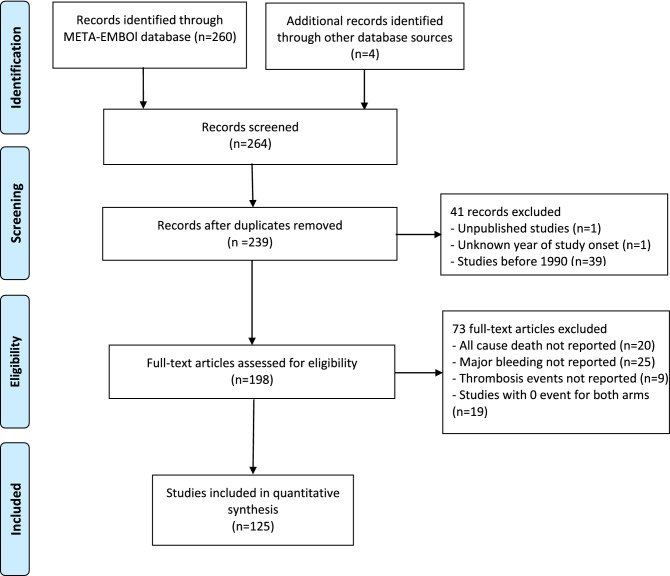
PRISMA flowchart. NCB, net clinical benefit.

The primary META-EMBOL database and the additional literature search identified 264 trials for review. Among them, there were 25 duplicated studies, 41 studies were excluded as they did not meet the year of publication criterion, and 73 articles were excluded after full-text review. Finally, a total of 125 eligible studies were included for data extraction (Fig. 1).

Among those included studies, 27 studies were conducted in the field of NVAF (114,689 patients), 27 focused on acute treatment of VTE (55,216 patients), 25 on thromboprophylaxis in patients hospitalized for medical conditions (69,022 patients), and 38 on major orthopedic and abdominal surgery (70,982 patients). Additionally, 8 studies (6372 patients) assessed thromboprophylaxis in cancer patients. A total of 79 studies were double-blind randomized controlled trials, 28 were open-label with blind adjudication, and 18 had an open-label design.

Treatments assessed in experimental arms were apixaban (k = 8), acenocoumarol (k = 2), betrixaban (k = 2), dabigatran (k = 8), rivaroxaban (k = 12), dalteparin (k = 4), edoxaban (k = 2), AZD0837 (k = 1), idrabiotaparinux (k = 2), fondaparinux (k = 10), aspirin (k = 5), dipyridamole (k = 2), nadroparin (k = 11), ximelagatran (k = 4), enoxaparin (k = 19), reviparin (k = 2), bemiparin (k = 1), semuloparin (k = 6), indobufen (k = 1), UFH (k = 2), tinzaparin (k = 1), other LMWH (k = 2), certoparin (k = 5), gemcitabine + dalteparin (k = 1), fraxiparin (k = 1), ardeparin (k = 2), and warfarin with/without aspirin (k = 9; Table 1).

**Table 1:** Studies characteristics and main antithrombotics for all the studied diseases.

Author, year	Study acronym	Design	Disease	Studied treatment	Comparison(s)	No. of patients
Koudstaal, 1993	EAFT	Open	AF	Warfarin adj stand INR 2.5–4.0, aspirin 300 mg	Placebo	1007
BÜller, 2008	AMADEUS	PROBE	AF	Idraparinux 2.5 mg od	Warfarin INR 2–3	4576
Connolly, 2006	ACTIVE W	Open	AF	Aspirin 75-100 mg + Clopidogrel 75 mg	VKA adj stand INR 2–3	6706
Connolly, 2011	AVERROES	DB	AF	Apixaban 5 mg bid	Aspirin 81 to 324 mg	5599
Connolly, 1991	CAFA	DB	AF	Warfarin adj stand INR 2–3	Placebo	378
Connolly, 2009	RE-LY	PROBE	AF	Dabigatran 110/150 mg bid	Warfarin INR 2–3	18,113
Diener, 1996	ESPS-2	DB	AF	Aspirin 25 mg bid, Dipyridamole 200 mg + Aspirin 25 mg bid, Dipyridamole 200 mg bid	Placebo	6602
Ezekowitz, 1992	SPINAF	DB	AF	Warfarin adj stand INR 1.4–2.8	Placebo	525
Giugliano, 2013	ENGAGE AF-TIMI	DB	AF	Edoxaban 30/60 mg od	Warfarin INR 2–3	21,105
Granger, 2011	ARISTOTLE	DB	AF	Apixaban 5 mg bid	Warfarin INR 2–3	18,201
Gullov, 1998	AFASAK II	Open	AF	Aspirin 300 mg od, Warfarin 1.25 mg + Asprin 300 mg od, warfarin	Warfarin adj stand INR 2–3	677
Halperin, 2005	SPORTIF V	DB	AF	Ximelagatran 36 mg bid	Warfarin INR 2–3	3922
Hellemons, 1999	PATAF	Open	AF	Aspirin 150 mg od	Acenocoumarol	729
Hori, 2012	J-ROCKET AF	DB	AF	Rivaroxaban 15 mg od	Warfarin INR 2–3	1280
Kistler, 1990	BAATAF	PROBE	AF	Warfarin adj stand INR 1.5–2.7	No treatment	420
Lip, 2009	NCT00684307	PROBE	AF	AZD0837 300 mg od	Warfarin INR 2–3	471
Mant, 2007	BAFTA	PROBE	AF	Warfarin adj stand INR 2–3	Aspirin 75 mg od	973
McBride, 1991	SPAF I	Open	AF	Aspirin 325 mg od, Warfarin adj stand INR 2–4.5	Placebo	1330
McBride, 1996	SPAF III	PROBE	AF	Warfarin adj low INR 1.2–1.5 + Aspirin 325 mg od	Warfarin adj stand INR 2–3	1044
Morocutti, 1997	SIFA	Open	AF	Indobufen 200 mg bid or 100 mg bid	Warfarin adj stand INR 2–3.5	916
Olsson, 2003	SPORTIF III	PROBE	AF	Ximelagatran 36 mg bid	Warfarin INR 2–3	3407
Pérez-Gomez, 2004	NASPEAFa	PROBE	AF	Triflusal 600 mg od + coumadin medium dose INR 1.25–2, Triflusal 600 mg od	Coumadin standard dose INR 2–3	714
Pérez-Gomez, 2004	NASPEAFb	PROBE	AF	Triflusal 600 mg od + coumadin medium dose INR 1.25–2	Triflusal 600 mg od	495
Patel, 2011	ROCKET AF	DB	AF	Rivaroxaban 20 mg od	Warfarin INR 2–3	14,264
Pengo, 2010	/	Open	AF	Warfarin adj low INR 1.5–2	Warfarin adj stand INR 2–3	267
Sato, 2006	JAST	Open	AF	Aspirin 150-200 mg od	No treatment	871
Connolly, 2013	EXPLORE-Xa	DB	AF	Betrixaban 40, 60, 80 mg od	Warfarin INR 2–3	254
Büller, 2012	CASSIOPEA	DB	PE	Idrabiotaparinux 3 mg ow	Warfarin INR 2–3	3202
Büller, 1997	COLOMBUS	Open	DVt, PE	Reviparin 6300 anti-Xa bid	UFH Bolus 5000 IU then 1250 IU/h	1012
Büller, 2010	EINSTEIN DVT	PROBE	DVT	Rivaroxaban 15 mg bid then 20 mg od	Enoxaparin 1 mg/kg bid + Warfarin INR 2–3	3449
Büller, 2008	EINSTEIN DVT	PROBE	DVT	Rivaroxaban 20 mg od	fraxiparin + VKA INR 2–3	273
Büller, 2012	EINSTEIN PE	PROBE	PE + / − DVT	Rivaroxaban 15 mg bid then 20 mg od	Enoxaparin 1 mg/kg bid + Warfarin or acenocoumarol INR 2–3	4833
Büller, 2011	EQUINOX	DB	DVT	Idrabiotaparinux 3 mg ow	Idrparinux 2.5 mg ow	757
Büller, 2013	HOKUSAI VTE	DB	DVt, PE	Edoxaban 60 mg od	Warfarin INR 2–3	8292
Büller, 2004	MATISSE DVT	DB	DVT	Fondaparinux 5 mg od	Enoxaparin 1 mg/kg bid	2205
Büller, 2003	MATISSE PE	PROBE	PE	Fondaparinux 5 mg od	UFH Bolus 5000 IU + 1250 IU/h aPTT	2213
Büller, 2007	VAN GOGH DVT	PROBE	DVT	Idraparinux 2.5 mg ow	Warfarin INR 2–3	2904
Büller, 2007	VAN GOGH PE	PROBE	PE	Idraparinux 2.5 mg ow	Warfarin INR 2–3	2215
Agnelli, 2013	AMPLIFY	DB	DVT, PE	Apixaban 10 mg, 5 mg bid	Enoxaparin 1 mg/kg then Warfarin INR 2–3	5395
Agnelli, 2013	AMPLIFY EXT	DB	DVT, PE	Apixaban 2.5 mg or 5 mg bid	Placebo	2486
Brandjes, 1992	/	DB	DVT	UFH Bolus 5000 IU then 1250 IU/h	Placebo	120
Hull, 1992	/	DB	DVT	LMWH 175 UI/kg od	UFH Bolus 5000 IU	432
Kearon, 2006	FIDO	PROBE	DVT, PE	LMWH 100 IU/kg	UFH 333 U/kg then 250 U/kg bid	708
Levine, 1996	/	PROBE	DVT	Enoxaparin 1 mg/kg bid	UFH Bolus 5000 IU then 20000 IU	500
Merli, 2001	/	PROBE	DVT, PE	Enoxaparin 1 mg/kg bid or 1.5 mg/kg od	UFH	900
Prandoni, 2004	GALILEI	PROBE	DVT, PE	Nadroparin 80 U/kg bid	UFH 4000 U + 12,500 U	720
Prandoni et al., 1992	/	Open	DVT	Nadroparin 0.5 ml bid	UFH Bolus 100 IU/kg then 35000 IU od	170
Schulman, 2009	RE-COVER	DB	DVT, PE	Dabigatran 150 mg bid	Warfarin INR 2–3	2539
Schulman, 2013	RE-COVER II	DB	DVT, PE	Dabigatran 150 mg bid	Warfarin INR 2–3	2568
Schulman, 2013	RE-MEDY	DB	DVT, PE	Dabigatran 150 mg bid	Warfarin INR 2–3	2866
Simonneau, 1997	THÉSÉE	PROBE	PE	Tinzaparin IU/kg od	UFH Bolus 50 IU/kg then 500 IU/kg od	612
Büller, 2007	VAN GOGH EXT	PROBE	DVT, PE	Idraparinux 2.5 mg ow	Placebo	1215
Eriksson, 2003	THRIVE I	PROBE	DVT	Enoxaparin 24 mg bid	Dalteparin 200U/kg + Warfarin INR 2–3	141
Fiessinger, 2005	THRIVE	DB	DVT + / − PE	Ximelagatran 36 mg bid	Warfarin INR 2–3	2528
Leizorovicz, 2004	PREVENT	DB	CHF	Dalteparin 5000 IU od	Placebo	3706
Lederle, 2006	/	DB	MP	Enoxaparin 4000 IU od	Placebo	280
Samama, 1999	MEDENOX	DB	CHF	Enoxaparin 4000 IU od	Placebo	738
Fraisse, 2000	/	DB	PD	Nadroparin 3800–5700 IU	Placebo	223
Mahe, 2005		DB	AMI	Nadroparin 2850 IU	Placebo	2474
Diener, 2006	PROTECT	DB	AIS	Certoparin 3000 IU od	UFH 5000 IU × 3	545
Riess, 2010	CERTIFY	DB	MP	Certoparin 3000 IU od	UFH 5000 IU × 3	3244
Schellong, 2010	CERTAIN	PROBE	MP	Certoparin 3000 IU od	UFH 7500 IU bid	337
Harenberg, 1990	/	DB	MP	Dalteparin 2500 IU	UFH 5000 IU × 3	166
Hillbom, 2002	/	DB	AIS	Enoxaparin 4000 IU od	UFH 5000 IU × 3	212
Sherman, 2007	PREVAIL	PROBE	AIS	Enoxaparin 4000 IU od	UFH 5000 IU bid	1762
Bergmann, 1996	EMSG	DB	AMI	Enoxaparin 2000 IU	UFH 5000 IU bid	442
Lechler, 1996	PRIME	DB	MP	Enoxaparin 4000 IU od	UFH 5000 IU × 3	959
Kleber, 2003	PRINCE	PROBE	SRD	Enoxaparin 4000 IU od	UFH 5000 IU × 3	665
Aquino, 1990	/	Open	MP	Nadroparin 2850 IU	UFH 10,000 or 15,000 IU	99
Manciet, 1990	APTE	DB	Elderly population	Nadroparin 2850 IU	UFH 5000 IU bid	256
Forette, 1995	/	Open	Elderly population	Nadroparin 2850 IU	UFH 5000 IU bid	295
Harenberg, 1996	HESIM	DB	MP	Nadroparin 2850 IU	UFH 5000 IU × 3	1590
Gardlund, 1996	/	Open	Infectious disease	UFH 5000 IU bid	No treatment	11,693
Cohen, 2006	ARTEMIS	DB	AMI	Fondaparinux 2.5 mg od	Placebo	849
Goldhaber, 2011	ADOPT	DB	AMI	Apixaban 2.5 mg bid	Enoxaparin 4000 IU od	4972
Cohen, 2013	MAGELLAN	DB	AMI	Rivaroxaban 10 mg od 10/35 days	Enoxaparin 4000 IU od	8101
Cohen, 2016	APEX	DB	AMI	Betrixaban 160 mg then 80 mg od	Placebo	7513
Hull, 2010	EXCLAIM	DB	MP	Enoxaparin 4000 IU od	Placebo	6085
Spyropoulos, 2018	MARINER	DB	MP	Rivaroxaban 10 mg or 7.5 mg od	Placebo	12,019
Levine, 1996	/	DB	TKR	Ardeparin 50 IU/kg bid	Placebo	246
Leclerc, 1992	/	DB	TKR	Enoxaparin 3000 IU bid	Placebo	131
Colwell, 1994	/	Open	HR	Enoxaparin 3000 IU bid or 4000 IU od	UFH 5000 IU × 3	610
Leyvraz A VALIDER, 1991	/	Open	TKR	Fraxiparin 41 IU/kg od then 62 IU/kg od	UFH × 3	409
Haas, 2006	/	DB	TKR + THR	Reviparin 4200 IU od	UFH 7500 IU bid	2018
Colwell, 1999	/	Open	Primary hip arthroplasty	Enoxaparin 3000 IU bid	Warfarin 7.5 mg INR 2–3	3011
Fitzgerald, 2001	/	PROBE	TKR	Enoxaparin 3000 IU bid	VKA INR 2–3	349
Leclerc, 1996	/	DB	TKR	Enoxaparin 3000 IU bid	VKA INR 2–3	670
Lassen, 2009	ADVANCE-1	DB	TKR	Apixaban 2.5 mg bid	Enoxaparin 3000 IU bid	3195
Lassen, 2010	ADVANCE-2	DB	TKR	Apixaban 2.5 mg bid	Enoxaparin 4000 IU od	3057
Lassen, 2010	ADVANCE-3	DB	THR	Apixaban 2.5 mg bid	Enoxaparin 4000 IU od	5407
Eriksson, 2007	RE-MODEL	DB	TKR	Dabigatran 150/220 mg	Enoxaparin 4000 IU od	2101
Eriksson, 2007	RE-NOVATE	DB	THR	Dabigatran 150/ 220 mg od	Enoxaparin 4000 IU od	3494
Eriksson, 2010	RE-NOVATE-2	DB	THR	Dabigatran 220 mg od	Enoxaparin 4000 IU od	2055
Ginsberg, 2008	RE-MOBILIZE	DB	TKR	Dabigatran 150/220 mg od	Enoxaparin 3000 IU bid	2615
Eriksson, 2008	RECORD 1	DB	THR	Rivaroxaban 10 mg od	Enoxaparin 4000 IU od	4541
Lassen, 2008	RECORD 3	DB	TKR	Rivaroxaban 10 mg od	Enoxaparin 4000 IU od	2531
Turpie, 2009	RECORD 4	DB	TKR	Rivaroxaban 10 mg od	Enoxaparin 3000 IU bid	3148
Bauer, 2001	PENTAMAKS	DB	MKS	Fondaparinux 2.5 mg od	Enoxaparin 3000 IU bid	1049
Eriksson, 2001	PENTHIFRA	DB	HFS	Fondaparinux 2.5-mg od	Enoxaparin 4000 IU od	1711
Lassen, 2002	EPHESUS	DB	HFS	Fondaparinux 2.5-mg od	Enoxaparin 4000 IU od	2309
Turpie, 2002	PENTATHLON	DB	HFS	Fondaparinux 2.5-mg od	Enoxaparin 3000 IU bid	2275
Heit, 2000	/	DB	THR	Ardeparin 100 IU/kg od	Placebo	1195
Comp, 2001	/	DB	TKR, THR	Enoxaparin 4000 IU od	Placebo	873
Eriksson, 2003	PENTHIFRA-Plus	DB	HFS	Fondaparinux 2.5 mg od	Placebo	656
Kakkar, 2008	RECORD2	DB	THR	Rivaroxaban 10 mg od	Enoxaparin 40 mg od	2509
Anderson, 2018	/	DB	THR, TKR	Rivaroxaban 10 mg od	Aspirin 81 mg od	3424
Lassen, 2012	SAVE-HIP1	DB	THR	Semuloparin 20 mg od	Enoxaparin 40 mg od	2326
Lassen, 2012	SAVE-HIP2	DB	HF	Semuloparin 20 mg od	Enoxaparin 40 mg od	1003
Lassen, 2012	SAVE-KNEE	DB	TKR	Semuloparin 20 mg od	Enoxaparin 30 mg bid	1150
Fisher, 2013	SAVE-HIP3	DB	Upper third of the femur	Semuloparin 20 mg od	Placebo	469
Ho, 1999	/	PROBE	MCS	Enoxaparin 4000 IU od	No treatment	303
Rasmussen, 2006	FAME	Open	MAS	Dalteparin 5000 IU od	No treatment	427
Bergqvist, 2002	ENOXACAN II	DB	MAS	Enoxaparin 4000 IU od	Placebo	501
Kakkar, 2010	CANBESURE	DB	MAS	Bemiparin 3500 IU od	Placebo	626
Turpie, 2007	APOLLO	DB	MAS	Fondaparinux 2.5 mg od	Placebo	1309
Agnelli, 2005	PEGASUS	DB	MAS	Fondaparinux 2.5 mg od	Dalteparin 5000 IU od	2927
Kakkar, 2013	SAVE-ABDO	DB	ML	Semuloparin 20 mg od	Enoxaparin 40 mg od	4352
Haas, 2012	TOPIC-1	DB	CP	Certoparin 3000 IU od	Placebo	353
Haas, 2012	TOPIC-2	DB	CP	Certoparin 3000 IU od	Placebo	547
Perry, 2010	PRODIGE	DB	CP	Dalteparin 5000 U od	Placebo	186
Maraveyas, 2012	FRAGEM	Open	CP	Gemcitabine	Dalteparin + Gemcitabine 200 IU/kg od then 150 IU/kg	123
Klerk, 2005	MALT	DB	CP	Nadroparin 9500 U/ml	Placebo	302
Agnelli, 2009	PROTECHT	DB	CP	Nadroparin 3800 IU od	Placebo	1150
van Doormaal, 2011	INPACT	PROBE	CP	Nadroparin	Placebo	503
Agnelli, 2012	SAVE-ONCO	DB	CP	Semuloparin 20 mg od	Placebo	3212

*AF* atrial fibrillation, *VTE* venous thromboembolism, *DVT* deep vein thrombosis, *PE* pulmonary embolism, *CHF* congestive heart failure, *MP* medical patients, *PD* pulmonary disease, *AMI* acute medical illness, *AIS* acute ischemic stroke, *SRD* severe respiratory disease, *TKR* total knee replacement, *THR* total hip replacement, *MKS* major knee surgery, *HFS* hip fracture surgery, *MAS* major abdominal surgery, *MCS* major colorectal surgery, *ML* major laparotomy, *CP* cancer patients, *DB* double-blind, *PROBE* prospective, randomized, open, blinded-endpoint.

### Surrogacy evaluation by clinical indication

**Figure 2:**
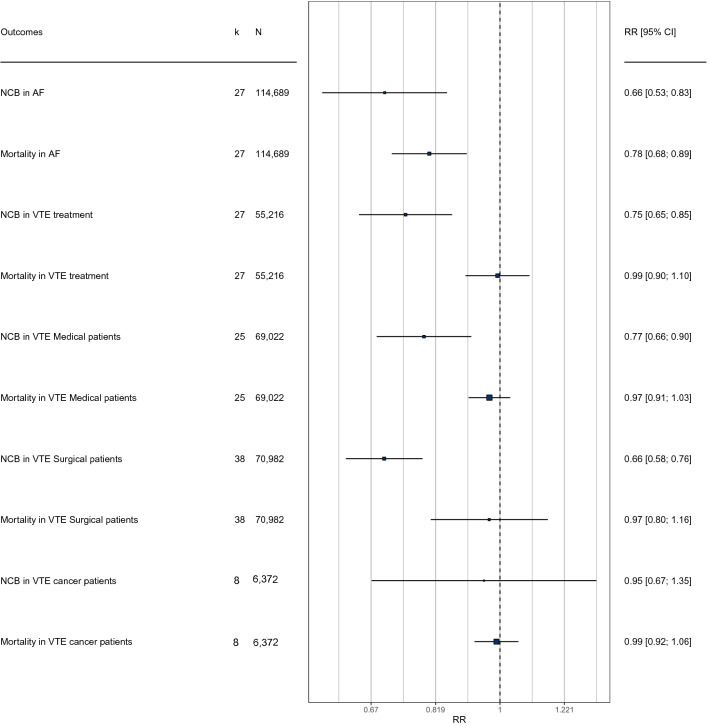
Summary of the meta-analysis and forest plot for all indications. Forest plot of treatment effects on all-cause mortality and net clinical benefit (NCB). The horizontal error bars show the 95% confidence interval (CI) of each relative risk (RR) based on the random-effect model. The square represents the RR. An RR of < 1 favors the experimental group, an RR = 1 indicates no difference in treatment effects, and an RR of > 1 indicates a harmful effect of the control group. AF = Atrial fibrillation, K = number of studies for each indication, MB = Major bleeding, N = Total number of the included patients, VTE = Venous thromboembolism.

Summary of the meta-analysis and forest plot for all indications are shown in Fig. 2, while the results of meta-analysis and forest plot for each disease separately are presented in Appendix A. The results are presented according to drug classes in Appendix C.

**Figure 3:**
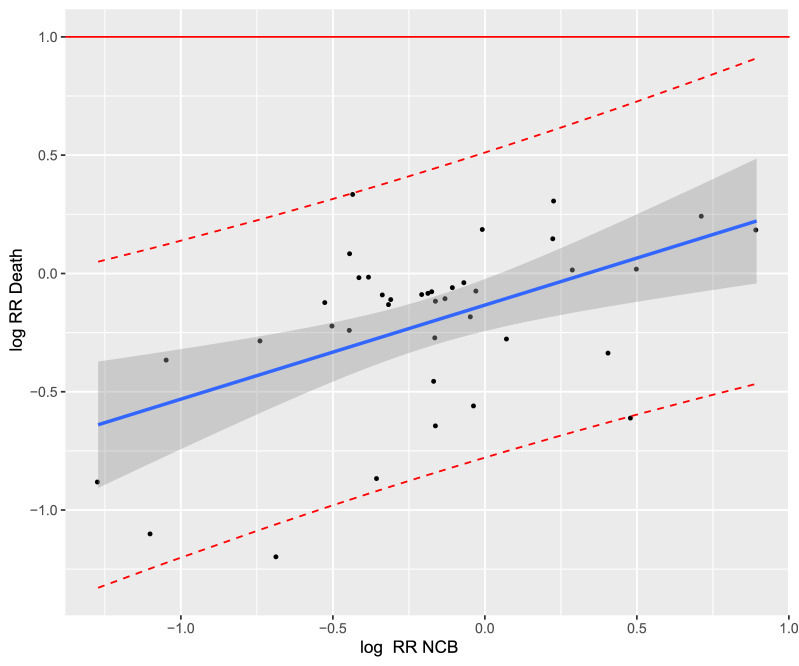
Trial-level association between treatment effects on net clinical benefit (NCB) and all-cause mortality in the treatment of non-valvular atrial fibrillation. The correlation was (Cor) = 0.62 with the linear regression model: "Log RR_Death_ = 0.44 × Log RR_NCB_ − 0.11”. Each study is represented by a circle. A log scale was used for the x-axis and y-axis. The solid blue line represents the regression line and the grey area represents the 95% confidence interval. The red dashed lines represent the upper and lower limits of the 95% prediction interval. RR, relative risk.

**Figure 4:**
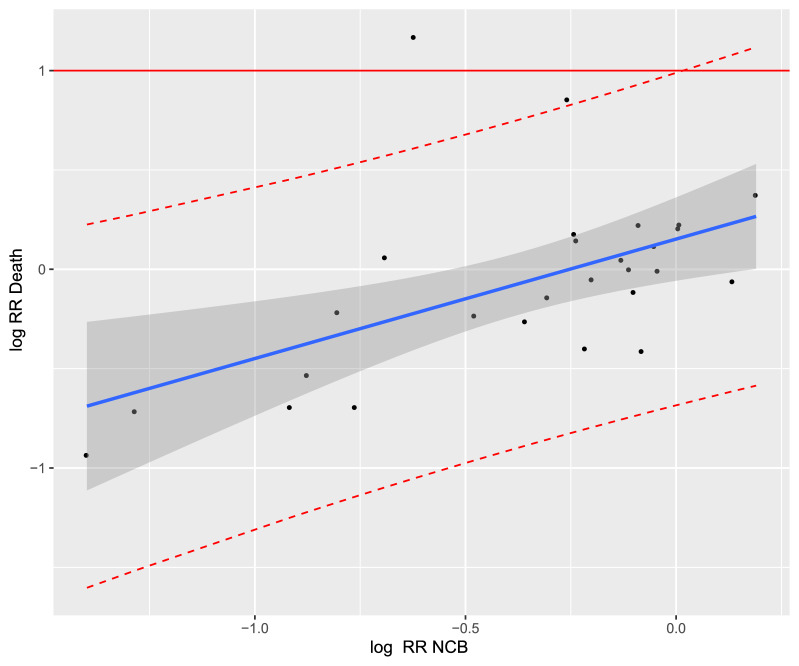
Trial-level association between treatment effects on net clinical benefit (NCB) and all-cause mortality in the treatment of acute venous thromboembolism. Cor = 0.24. The corresponding linear regression model was "Log RR_Death_ = 0.6 × Log RR_NCB_ + 0.15”. Each study is represented by a circle. A log scale was used for the x-axis and y-axis. The solid blue line represents the regression line and the grey area represents the 95% confidence interval. The red dashed lines represent the upper and lower limits of the 95% prediction interval. RR, relative risk.

The coefficient of determination of the treatment effects was the highest for NVAF studies (R^2^_trial_ = 0.41, 95% CI [0.03; 0.48]; Fig. 3). For acute VTE studies, the coefficient of determination was R^2^_trial_ = 0.30 (95% CI [0.04; 0.84]; Fig. 4). Thus, in both NVAF and acute VTE studies, the correlation between NCB and all-cause mortality was weak.

**Figure 5:**
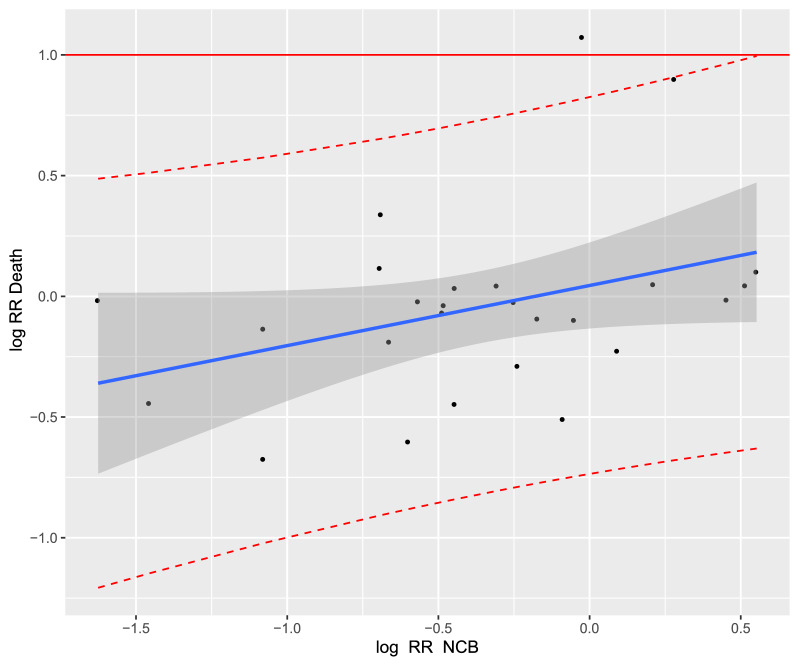
Trial-level association between treatment effects on net clinical benefit (NCB) and all-cause mortality in the treatment of the prevention of VTE in medical patients. Cor = 0.32 and the linear regression model was "Log RR_Death_ = 0.26 × Log RR_NCB_ + 0.05”. Each study is represented by a circle. A log scale was used for the x-axis and y-axis. The solid blue line represents the regression line and the grey area represents the 95% confidence interval. The red dashed lines represent the upper and lower limits of the 95% prediction interval. RR, relative risk.

**Figure 6:**
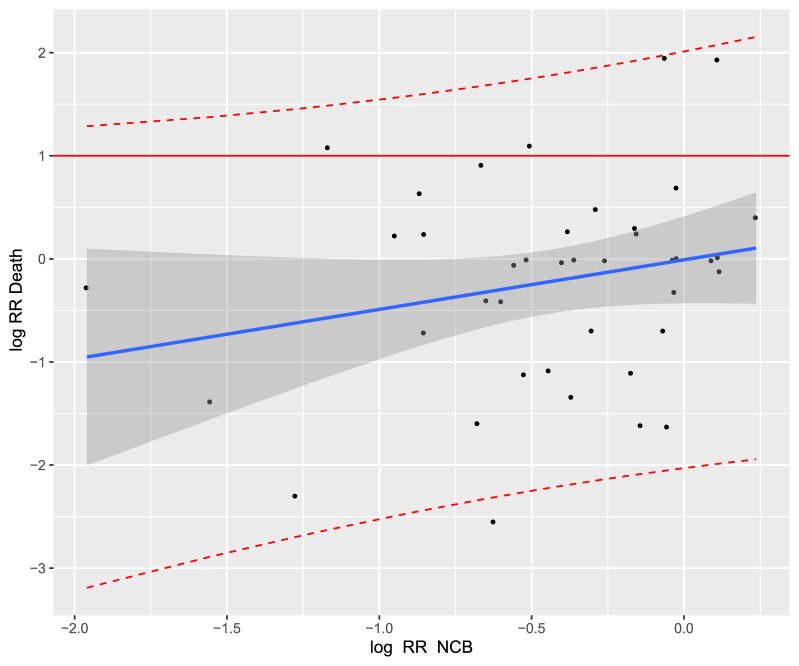
Trial-level association between treatment effects on net clinical benefit (NCB) and all-cause mortality in the treatment of the major orthopedic and abdominal surgery. Cor = 0.24 with the linear regression model "Log RR_Death_ = 0.49 × Log RR_NCB_ − 0.05". Each study is represented by a circle. A log scale was used for the x-axis and y-axis. The solid blue line represents the regression line and the grey area represents the 95% confidence interval. The red dashed lines represent the upper and lower limits of the 95% prediction interval. RR, relative risk.

**Figure 7:**
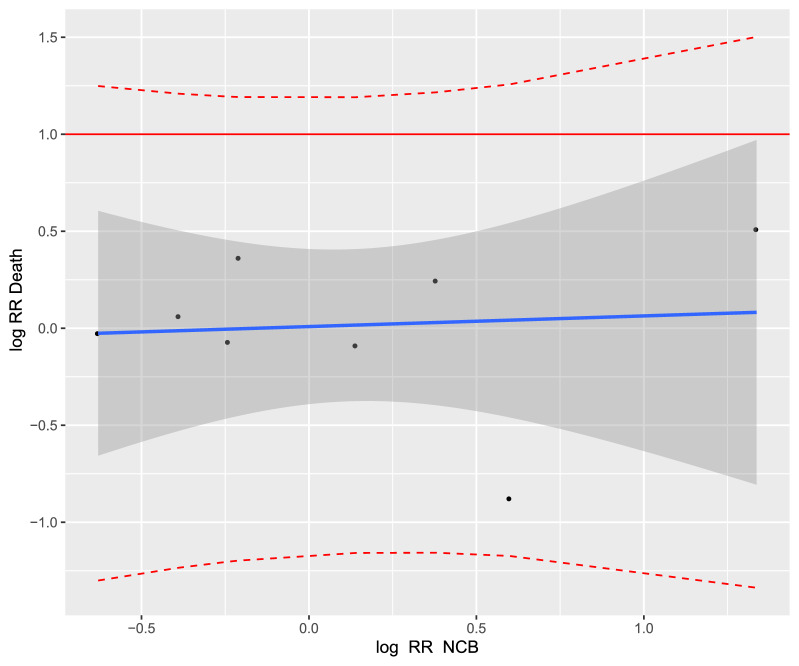
Trial-level association between treatment effects on net clinical benefit (NCB) and all-cause mortality in the treatment of the other prevention in cancer patients. Cor = 0.41 and the linear regression model: "Log RR_Death_ = -0.05 × Log RR_NCB_ + 0.01". Each study is represented by a circle. A log scale was used for the x-axis and y-axis. The solid blue line represents the regression line and the grey area represents the 95% confidence interval. The red dashed lines represent the upper and lower limits of the 95% prediction interval. RR, relative risk.

Regarding the coefficient of determination for studies investigating the prevention of VTE, there was no correlation between NCB and all-cause mortality for medical patients (R^2^_trial_ = 0.12, 95% CI [0.00; 0.36]; Fig. 5), neither for surgical patients (R^2^_trial_ = 0.05, 95% CI [0.00; 0.23]; Fig. 6), nor for cancer patients (R^2^_trial_ = 0.006, 95% CI [0.00; 1.00]; Fig. 7).

### Surrogate threshold effect (STE)

Considering the lower limit of the prediction interval of the treatment effect on the surrogate endpoint in the prevention of VTE, treatment of VTE, and treatment of NVAF, the STE could not be determined and calculated for all the indications previously mentioned.

### Sensitivity and post-hoc analyses

The coefficients of determination for double-blind clinical trials only and for those evaluating DOAC for the acute treatment of VTE only were higher than the primary analysis, but their wide confidence intervals did not support significant differences (Results of primary and sensitivity analysis are shown in details in Table 2). Similarly, the linear regression found no significant correlation between the overall death rate and the relative risk reduction of all-cause mortality (Figures of forest plot and GGPLOT are shown in Appendix B).

**Table 2:** Primary and sensitivity analysis for all the studied diseases.

		Primary outcomes			Sensitivity analysis (only DB)				Sensitivity analysis (only DOAC)		
		RR (95% CI)	R^2^ (95% CI)	*p* Value	RR (95% CI)	R^2^ (95% CI)		*p* Value	RR (95% CI)	R^2^ (95% CI)	*p* Value
NVAF	NCB	0.66 (0.53; 0.83)	0.41 (0.03; 0.48)	0.0003	0.61 (0.45; 0.83)		0.30 (0.0002, 0.71)	0.08	0.66 [0.47; 0.94)	0.37 (0.007, 0.88)	0.1
	ACM	0.78 (0.68; 0.89)			0.89 (0.86; 0.93)				0.79 (061; 1.02)		
Acute VTE	NCB	0.73 (0.63; 0.85)	0.30 (0.04; 0.84)	0.003	0.63 (0.48; 0.83)		0.75 (0.006 ; 0.98)	0.0002	0.74 (0.61; 0.90)	0.50 (0.02 ; 0.91)	0.001
	ACM	0.98 (088; 1.10)			0.89 (0.75; 1.05)				1.04 (0.94; 1.15)		
Thrombop. in medical patients	NCB	0.77 (0.66; 0.90)	0.12 (0.0005; 0.36)	0.08	0.81 (0.67;0.98)		0.11 (0.0003, 0.46)	0.17	0.87 (0.69; 1.09)	0.04 (0.0002, 0.70)	0.59
	ACM	0.97 (0.91; 1.03)			0.98 (0.91;1.06)				0.97 (0.88; 1.06)		
Thrombop. in major orthopedic surgery	NCB	0.66 (0.58; 0.76)	0.05 (0.0002; 0.23)	0.16	0.65 (0.56; 0.76)		0.07 (0.0003, 0.28)	0.16	0.66 (0.55; 0.79)	0.08 (0.0004, 0.3)	0.15
	ACM	0.97 (0.80; 1.16)			0.95 (0.77; 1.17)				0.98 (0.81; 1.22)		
Thrombop. in cancer patients	NCB	0.98 (0.67; 1.44)	0.006 (0.0001; 1)	0.84	0.75 (0.56; 1.01)		0.26 (0.0000; 1.00)	0.30	/	/	/
	ACM	0.98 (0.92; 1.04)			0.99 (0.93; 1.06)				/		

*ACM* all-cause mortality, *CI* confidence interval, *DB* double-blind, *DOAC* direct oral anticoagulants, *NCB* net clinical benefit, *NVAF* non-valvular atrial fibrillation, *Thrombop*. thromboprophylaxis, *RR* relative risk, *R*^2^ coefficient of determination, *VTE* venous thromboembolism.

## Discussion

The objective of this study was to describe the relation between NCB and all-cause mortality to validate this outcome as a surrogate endpoint in NVAF and VTE trials using meta-regression. While the coefficient of determination R^2^_trial_ was low for acute VTE and NVAF studies, the correlation between the NCB and all cause-mortality was very weak. Additionally, no correlation was observed in prevention studies for which the R^2^_trial_ were negligible. These results were also consistent irrespective of experimental treatments and study design. Taken together, these results do not support the use of NCB as a surrogate endpoint for all-cause mortality in NVAF and VTE trials.

The limited association between NCB and all-cause mortality reduction may be explained by several factors. First, major bleeding and thrombosis may not lead systematically to death even though they are morbid outcomes. Indeed, in a post-hoc analysis of the ROCKET-AF trial comparing rivaroxaban and warfarin in NVAF^26^, only 1 in 10 deaths has been related to MB and ischemic stroke. In addition, a meta-analysis that analyzed the causes of death in patients receiving DOAC or warfarin in NVAF has reported that ischemic strokes and fatal bleedings were responsible for a minority (6%) of all death, while the main cause of death in NVAF appeared to be related to sudden cardiac death, heart failure, and myocardial infarction rather than outcomes targeted by study protocols^27^. Thus, anticoagulants have limited impacts on events ultimately leading to deaths among NVAF patients. Indeed, the low incidence of VTE and bleeding might be due to the improvement in patient management strategy^28^. Of note, the results herein did not show a relationship between the crude mortality rate and mortality risk reduction related to anticoagulant exposure. Thus, a lack of power related to low event incidence is unlikely.

Similarly, cancer has been found to be the most common cause of death (42%) in a meta-analysis of seven randomized trials evaluating DOAC for the treatment of VTE^29^, whereas recurrent VTE and fatal bleeding have been estimated to be responsible for only 20% and 6% of deaths, respectively.

Furthermore, a study combining the results of ACTIVE and RE-LY trials^30,31^ has calculated the NCB according to the relative weights of different events, and has reported that the clinical importance of major bleeding events, except hemorrhagic stroke, was less than that of ischemic stroke. Indeed, the adjusted hazard ratio of death after a hemorrhagic stroke, ischemic stroke, subdural hemorrhage, and major extracranial bleeding were highly different one from another (26.92, 8.33, 6.89, and 5.23, respectively). Hemorrhagic stroke has been reported to increase the risk of death by 3.29-fold per 100 patient-years compared to ischemic stroke^30^. Consequently, the relative importance and the clinical impact of major bleeding and thrombotic events are not similar and do not have the same weight and incidence^32^.

The European network for Health Technology Assessment does not recommend the use of a composite endpoint as a principal outcome measure when a suitable single primary endpoint is available, especially when the combined primary outcomes have different weights^33^. Therefore, the limited correlation between NCB and all-cause mortality found herein and the absence of recommendation from the regulatory agencies regarding the use of NCB in NVAF and VTE studies argue against the use of NCB as a primary outcome in randomized control trials.

Some limitations of the present study need to be noted. First, one trial identified in the database META EMBOL was not published and therefore not included in the present study, but it is unlikely that the inclusion of this trial would change the correlation between NCB and overall mortality found herein. Second, the number of patients, the experimental designs, the experimental and control groups, and the definitions of outcomes were variable between studies, which might have affected the statistical results. Additionally, only studies that measured the three outcomes (MB, recurrent ischemic/thrombotic event, and all-cause mortality) were included, without the composite or other outcomes, and so, the number of studies included was reduced. Finally, NCB was estimated in the present study by the sum of MB and thrombosis for all patients within each study, and not for each patient individually. This alternative calculation may have led to different results.

## Conclusion

A weak correlation between NCB and all cause-mortality was found in studies investigating NVAF and acute VTE, whereas no correlation was observed in clinical situations where the mortality rate was low. Therefore, using the NCB should not be considered as a validated surrogate outcome of all-cause mortality in NVAF, acute VTE, and VTE prevention trials.

## Supplementary Information

Supplementary Information 1.

Supplementary Information 2.

Supplementary Information 3.

Supplementary Information 4.

Supplementary Information 5.
