# Supplementary material for: Meta-regression of randomized control trials with antithrombotics: weak correlation between net clinical benefit and all cause-mortality
Source: Sci Rep. 2021 Jul 19;11:14728. doi: 10.1038/s41598-021-94160-1 (PMC8290002; doi:10.1038/s41598-021-94160-1)

Appendix A - Forest plot of relative risks (RR) for all-cause mortality and net clinical benefit for non-valvular atrial fibrillation (NVAF), acute venous thromboembolism (VTE), and prevention of VTE

A.1. Atrial fibrillation, outcome: All-cause mortality

A relative risk (RR) > 1 mean that the experimental arm was associated with more mortality than the control arm

Study	Experimental Events	Experimental Total	Control Events	Control Total	RR	95%−CI	Weight
Koudstaal − EAFT	143.0	629	143.0	378	0.60	[0.50; 0.73]	5.2%
Büller − AMADEUS	62.0	2283	61.0	2293	1.02	[0.72; 1.45]	4.2%
Connolly − ACTIVE W	159.0	3335	158.0	3371	1.02	[0.82; 1.26]	5.1%
Connolly − AVERROES	111.0	2808	140.0	2791	0.79	[0.62; 1.01]	4.9%
Connolly − CAFA	10.0	187	8.0	191	1.28	[0.52; 3.16]	1.6%
Connolly − RE−LY	884.0	12091	974.0	6022	0.45	[0.41; 0.49]	5.7%
Diener − ESPS−2	555.0	4953	202.0	1649	0.91	[0.79; 1.06]	5.5%
Ezekowitz − SPINAF	15.0	260	22.0	265	0.69	[0.37; 1.31]	2.5%
Giugliano − ENGAGE AF−TIMI	1510.0	14069	839.0	7036	0.90	[0.83; 0.97]	5.7%
Granger − ARISTOTLE	603.0	9120	669.0	9081	0.90	[0.81; 1.00]	5.7%
Gullov − AFASAK II	25.0	507	17.0	170	0.49	[0.27; 0.89]	2.7%
Halperin − SPORTIF V	116.0	1960	123.0	1962	0.94	[0.74; 1.21]	4.9%
Hellemons − PATAF	61.0	410	95.0	319	0.50	[0.38; 0.67]	4.6%
Hori − J−ROCKET AF	7.0	640	5.0	640	1.40	[0.45; 4.39]	1.1%
Kistler − BAATAF	11.0	212	26.0	208	0.42	[0.21; 0.82]	2.3%
Lip	0.5	151	2.5	318	0.42	[0.02; 8.72]	0.2%
Mant − BAFTA	107.0	488	108.0	485	0.98	[0.78; 1.25]	4.9%
McBride − SPAF I	45.0	762	58.0	568	0.58	[0.40; 0.84]	4.0%
McBride − SPAF III	42.0	521	35.0	523	1.20	[0.78; 1.86]	3.6%
Morocutti − SIFA	32.0	454	35.0	462	0.93	[0.59; 1.48]	3.4%
Olsson − SPORTIF III	78.0	1704	79.0	1703	0.99	[0.73; 1.34]	4.5%
Pérez−Gomez − NASPEAFa	26.0	479	35.0	235	0.36	[0.22; 0.59]	3.3%
Pérez−Gomez − NASPEAFb	12.0	236	23.0	259	0.57	[0.29; 1.12]	2.3%
Patel − ROCKET AF	208.0	7111	250.0	7125	0.83	[0.70; 1.00]	5.3%
Pengo	78.0	135	70.0	132	1.09	[0.88; 1.35]	5.1%
Sato − JAST	10.0	426	9.0	445	1.16	[0.48; 2.83]	1.6%
Connolly − EXPLORE−Xa	1.0	381	1.0	127	0.33	[0.02; 5.29]	0.2%
**Random effects model**		**66312**		**48758**	**0.78**	**[0.68; 0.89]**	**100.0%**

Heterogeneity: $I^2 = 89\%$, $\tau^2 = 0.08$, $p < 0.01$

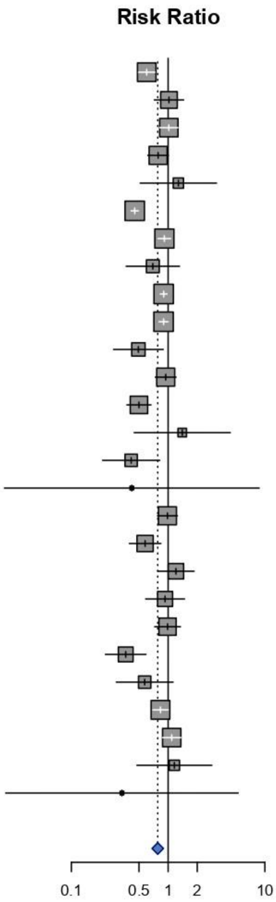

A.2. Atrial fibrillation, outcome: Net clinical benefit

A relative risk (RR) > 1 mean that the experimental arm was associated with more NCB than the control arm

Study	Experimental Events	Experimental Total	Control Events	Control Total	RR	95%−CI	Weight
Koudstaal − EAFT	127.0	629	147.0	378	0.52	[0.42; 0.63]	4.4%
Büller − AMADEUS	92.0	2283	56.0	2293	1.65	[1.19; 2.29]	4.2%
Connolly − ACTIVE W	201.0	3335	152.0	3371	1.34	[1.09; 1.64]	4.4%
Connolly − AVERROES	93.0	2808	144.0	2791	0.64	[0.50; 0.83]	4.3%
Connolly − CAFA	6.0	187	3.0	191	2.04	[0.52; 8.05]	1.6%
Connolly − RE−LY	1014.0	12091	1212.0	6022	0.42	[0.39; 0.45]	4.6%
Diener − ESPS−2	627.0	4953	771.0	1649	0.27	[0.25; 0.30]	4.5%
Ezekowitz − SPINAF	10.0	260	29.0	265	0.35	[0.17; 0.71]	3.1%
Giugliano − ENGAGE AF−TIMI	1313.0	14069	1682.0	7036	0.39	[0.37; 0.42]	4.6%
Granger − ARISTOTLE	526.0	9120	712.0	9081	0.74	[0.66; 0.82]	4.5%
Gullov − AFASAK II	38.0	507	42.0	170	0.30	[0.20; 0.45]	4.0%
Halperin − SPORTIF V	117.0	1960	130.0	1962	0.90	[0.71; 1.15]	4.3%
Hellemons − PATAF	40.0	410	43.0	319	0.72	[0.48; 1.09]	4.0%
Hori − J−ROCKET AF	37.0	640	57.0	640	0.65	[0.44; 0.97]	4.0%
Kistler − BAATAF	4.0	212	14.0	208	0.28	[0.09; 0.84]	2.1%
Lip	1.0	151	4.0	318	0.53	[0.06; 4.67]	0.8%
Mant − BAFTA	46.0	488	69.0	485	0.66	[0.47; 0.94]	4.1%
McBride − SPAF I	43.0	762	77.0	568	0.42	[0.29; 0.59]	4.1%
McBride − SPAF III	56.0	521	23.0	523	2.44	[1.53; 3.91]	3.8%
Morocutti − SIFA	22.5	454	23.5	462	0.97	[0.55; 1.71]	3.5%
Olsson − SPORTIF III	65.0	1704	95.0	1703	0.68	[0.50; 0.93]	4.2%
Pérez−Gomez − NASPEAFa	24.0	479	29.0	235	0.41	[0.24; 0.68]	3.6%
Pérez−Gomez − NASPEAFb	22.0	236	25.0	259	0.97	[0.56; 1.67]	3.6%
Patel − ROCKET AF	579.0	7111	607.0	7125	0.96	[0.86; 1.07]	4.5%
Pengo	23.0	135	35.0	132	0.64	[0.40; 1.03]	3.8%
Sato − JAST	24.0	426	20.0	445	1.25	[0.70; 2.24]	3.5%
Connolly − EXPLORE−Xa	5.5	381	5.5	127	0.33	[0.10; 1.07]	2.0%
**Random effects model**		**66312**		**48758**	**0.66**	**[0.53; 0.83]**	**100.0%**

Heterogeneity: $I^2 = 96\%$, $\tau^2 = 0.27$, $p < 0.01$

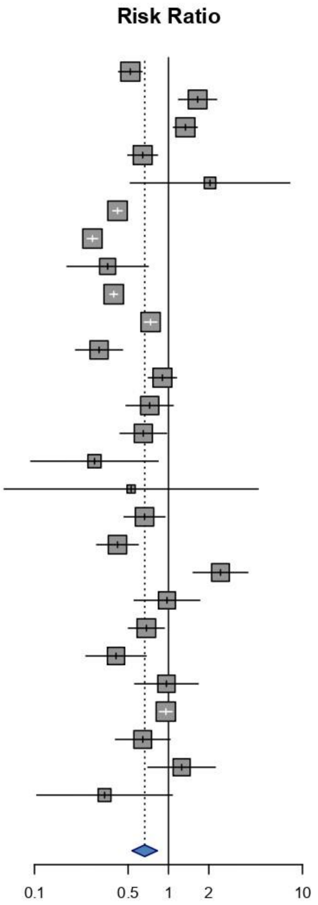

A.3. Acute treatment of venous thromboembolism, outcome: all-cause mortality.

A relative risk (RR) > 1 mean that the experimental arm was associated with more mortality than the control arm

Study	Experimental Events	Experimental Total	Control Events	Control Total	Risk Ratio	RR	95%−CI	Weight
Büller − CASSIOPEA	89.0	1599	84.0	1603		1.06	[0.79; 1.42]	9.4%
Büller − COLOMBUS	36.0	501	39.0	511		0.94	[0.61; 1.46]	5.0%
Büller − EINSTEIN DVT	38.0	1731	49.0	1718		0.77	[0.51; 1.17]	5.4%
Büller − EINSTEIN DVT dose ranging	4.0	136	5.0	137		0.81	[0.22; 2.94]	0.7%
Büller − EINSTEIN PE	58.0	2420	50.0	2413		1.16	[0.80; 1.68]	6.4%
Büller − EQUINOX	11.0	386	18.0	371		0.59	[0.28; 1.23]	2.0%
Büller − HOKUSAI VTE	132.0	4143	126.0	4149		1.05	[0.83; 1.33]	12.1%
Büller − MATISSE DVT	41.0	1098	33.0	1107		1.25	[0.80; 1.97]	4.7%
Büller − MATISSE PE	57.0	1103	48.0	1110		1.20	[0.82; 1.74]	6.4%
Büller − VAN GOGH DVT	55.0	1452	44.0	1452		1.25	[0.85; 1.85]	6.0%
Büller − VAN GOGH PE	64.0	1095	45.0	1120		1.45	[1.00; 2.11]	6.5%
Agnelli − AMPLIFY	41.0	2691	52.0	2704		0.79	[0.53; 1.19]	5.7%
Agnelli − AMPLIFY EXT	11.0	1657	14.0	829		0.39	[0.18; 0.86]	1.7%
Brandjes	1.0	60	2.0	60		0.50	[0.05; 5.37]	0.2%
Hull	10.0	213	21.0	219		0.49	[0.24; 1.01]	2.0%
Kearon − FIDO	22.0	353	18.0	355		1.23	[0.67; 2.25]	2.8%
Levine	11.0	247	17.0	253		0.66	[0.32; 1.39]	2.0%
Merli	18.0	610	9.0	290		0.95	[0.43; 2.09]	1.7%
Prandoni − GALILEI	12.0	360	12.0	360		1.00	[0.46; 2.20]	1.7%
Prandoni et al	6.0	85	12.0	85		0.50	[0.20; 1.27]	1.3%
Schulman − RE−COVER	21.0	1274	21.0	1265		0.99	[0.55; 1.81]	2.9%
Schulman − RE−COVER II	29.0	1279	26.0	1289		1.12	[0.67; 1.90]	3.7%
Schulman − RE−MEDY	17.0	1435	19.0	1431		0.89	[0.47; 1.71]	2.5%
Simonneau − THÉSÉE	12.0	304	14.0	308		0.87	[0.41; 1.85]	1.9%
Büller − VAN GOGH EXT	9.0	594	4.0	621		2.35	[0.73; 7.60]	0.8%
Eriksson − THRIVE I	1.5	68	0.5	73		3.22	[0.13; 77.72]	0.1%
Fiessinger − THRIVE	28.0	1240	42.0	1249		0.67	[0.42; 1.08]	4.4%
**Random effects model**		**28134**		**27082**		**0.99**	**[0.90; 1.10]**	**100.0%**

Heterogeneity: $I^2 = 18\%$, $\tau^2 < 0.01$, $p = 0.20$

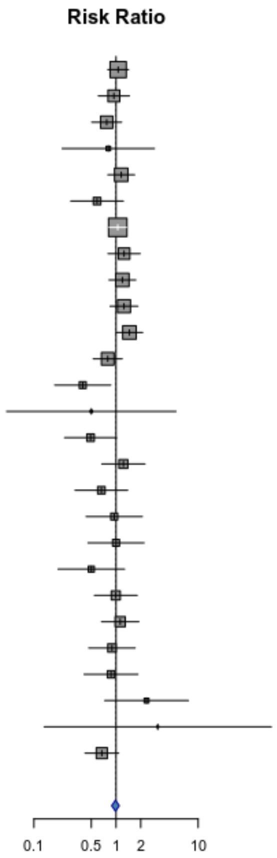

A.4. Acute treatment of venous thromboembolism, outcome: Net clinical benefit

A relative risk (RR) > 1 mean that the experimental arm was associated with more NCB than the control arm

Study	Experimental Events	Experimental Total	Control Events	Control Total	RR	95%-CI	Weight
Büller − CASSIOPEA	68	1599	136	1603	0.50	[0.38; 0.67]	5.1%
Büller − COLOMBUS	37	501	33	511	1.14	[0.73; 1.80]	4.0%
Büller − EINSTEIN DVT	50	1731	71	1718	0.70	[0.49; 1.00]	4.7%
Büller − EINSTEIN DVT dose ranging	4	136	9	137	0.45	[0.14; 1.42]	1.4%
Büller − EINSTEIN PE	76	2420	96	2413	0.79	[0.59; 1.06]	5.1%
Büller − EQUINOX	13	386	30	371	0.42	[0.22; 0.79]	3.0%
Büller − HOKUSAI VTE	186	4143	212	4149	0.88	[0.73; 1.06]	5.7%
Büller − MATISSE DVT	71	1098	71	1107	1.01	[0.73; 1.39]	4.9%
Büller − MATISSE PE	64	1103	82	1110	0.79	[0.57; 1.08]	4.9%
Büller − VAN GOGH DVT	54	1452	59	1452	0.92	[0.64; 1.31]	4.6%
Büller − VAN GOGH PE	52	1095	44	1120	1.21	[0.82; 1.79]	4.4%
Agnelli − AMPLIFY	74	2691	120	2704	0.62	[0.47; 0.82]	5.1%
Agnelli − AMPLIFY EXT	39	1657	79	829	0.25	[0.17; 0.36]	4.5%
Brandjes	6	60	15	60	0.40	[0.17; 0.96]	2.0%
Hull	7	213	26	219	0.28	[0.12; 0.62]	2.3%
Kearon − FIDO	17	353	17	355	1.01	[0.52; 1.94]	2.9%
Levine	18	247	20	253	0.92	[0.50; 1.70]	3.1%
Merli	31	610	18	290	0.82	[0.47; 1.44]	3.4%
Prandoni − GALILEI	17	360	19	360	0.89	[0.47; 1.69]	3.0%
Prandoni et al	7	85	15	85	0.47	[0.20; 1.09]	2.1%
Schulman − RE−COVER	54	1274	56	1265	0.96	[0.66; 1.38]	4.6%
Schulman − RE−COVER II	49	1279	52	1289	0.95	[0.65; 1.39]	4.5%
Schulman − RE−MEDY	39	1435	43	1431	0.90	[0.59; 1.39]	4.2%
Simonneau − THÉSÉE	8	304	11	308	0.74	[0.30; 1.81]	2.0%
Büller − VAN GOGH EXT	17	594	23	621	0.77	[0.42; 1.43]	3.1%
Eriksson − THRIVE I	3	68	6	73	0.54	[0.14; 2.06]	1.1%
Fiessinger − THRIVE	40	1240	50	1249	0.81	[0.54; 1.21]	4.3%
**Random effects model**		**28134**		**27082**	**0.75**	**[0.65; 0.86]**	**100.0%**

Heterogeneity: I^2 = 67%, τ^2 = 0.10, p < 0.01

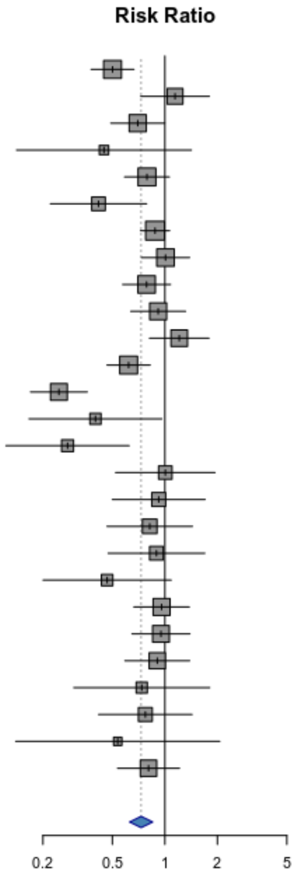

A.5. Thromboprophylaxis in patients hospitalized for medical conditions, outcome: All-cause mortality

A relative risk (RR) > 1 mean that the experimental arm was associated with more mortality than the control arm

Study	Experimental Events	Experimental Total	Control Events	Control Total	RR	95%−CI	Weight
Leizorovicz − PREVENT	107	1856	103	1850	1.04	[0.80; 1.35]	6.0%
Lederle	36	140	32	140	1.12	[0.74; 1.70]	2.4%
Samama − MEDENOX	41	367	50	371	0.83	[0.56; 1.22]	2.8%
Fraisse	8	109	8	114	1.05	[0.41; 2.69]	0.5%
Mahe	124	1230	128	1244	0.98	[0.78; 1.24]	7.6%
Diener − PROTECT	21	272	15	273	1.41	[0.74; 2.67]	1.0%
Riess − CERTIFY	66	1626	72	1618	0.91	[0.66; 1.26]	3.9%
Schellong − CERTAIN	9	163	15	174	0.64	[0.29; 1.42]	0.6%
Harenberg	3	84	1	82	2.93	[0.31; 27.58]	0.1%
Hillbom	21	106	28	106	0.75	[0.46; 1.23]	1.7%
Sherman − PREVAIL	100	884	103	878	0.96	[0.74; 1.25]	6.2%
Bergmann − EMSG	7	217	8	225	0.91	[0.33; 2.46]	0.4%
Lechler − PRIME	7	477	11	482	0.64	[0.25; 1.64]	0.5%
Kleber − PRINCE	9	332	15	333	0.60	[0.27; 1.36]	0.6%
Aquino	1	49	2	50	0.51	[0.05; 5.45]	0.1%
Manciet − APTE	8	129	8	127	0.98	[0.38; 2.54]	0.5%
Forette	6	146	7	149	0.87	[0.30; 2.54]	0.4%
Harenberg − HESIM	23	810	9	780	2.46	[1.15; 5.28]	0.7%
Gardlund	304	5776	333	5917	0.94	[0.80; 1.09]	18.1%
Cohen − ARTEMIS	14	429	25	420	0.55	[0.29; 1.04]	1.0%
Goldhaber − ADOPT	131	2484	133	2488	0.99	[0.78; 1.25]	7.5%
Cohen − MAGELLAN	231	4050	224	4051	1.03	[0.86; 1.23]	13.0%
Cohen − APEX	210	3716	215	3716	0.98	[0.81; 1.17]	12.1%
Hull − EXCLAIM	148	2975	142	2988	1.05	[0.84; 1.31]	8.2%
Spyropoulos − MARINER	71	6007	89	6012	0.80	[0.59; 1.09]	4.3%
**Random effects model**		**34434**		**34588**	**0.97**	**[0.91; 1.03]**	**100.0%**

Heterogeneity: $I^2 = 0\%$, $\tau^2 = 0$, $p = 0.72$

Test for overall effect: $z = -1.07$ ($p = 0.28$)

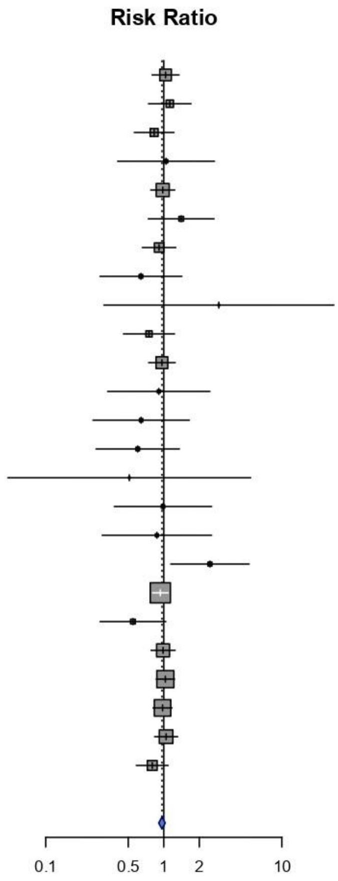

A.6. Thromboprophylaxis in patients hospitalized for medical conditions, outcome: Net clinical benefit

A relative risk (RR) > 1 mean that the experimental arm was associated with more NCB than the control arm

Study	Experimental Events	Experimental Total	Control Events	Control Total	RR	95%−CI	Weight
Leizorovicz − PREVENT	45	1856	70	1850	0.64	[0.44; 0.93]	5.9%
Lederle	7	140	14	140	0.50	[0.21; 1.20]	2.3%
Samama − MEDENOX	25	367	49	371	0.52	[0.33; 0.82]	5.0%
Fraisse	19	109	27	114	0.74	[0.44; 1.24]	4.4%
Mahe	23	1230	41	1244	0.57	[0.34; 0.94]	4.5%
Diener − PROTECT	3	272	6	273	0.50	[0.13; 1.99]	1.1%
Riess − CERTIFY	121	1626	143	1618	0.84	[0.67; 1.06]	7.4%
Schellong − CERTAIN	15	163	25	174	0.64	[0.35; 1.17]	3.8%
Harenberg	1	84	1	82	0.98	[0.06; 15.35]	0.3%
Hillbom	41	106	52	106	0.79	[0.58; 1.07]	6.5%
Sherman − PREVAIL	79	884	127	878	0.62	[0.47; 0.81]	7.0%
Bergmann − EMSG	11	217	12	225	0.95	[0.43; 2.11]	2.6%
Lechler − PRIME	3	477	13	482	0.23	[0.07; 0.81]	1.3%
Kleber − PRINCE	21	332	23	333	0.92	[0.52; 1.62]	4.0%
Aquino	1	49	3	50	0.34	[0.04; 3.16]	0.5%
Manciet − APTE	1	129	5	127	0.20	[0.02; 1.66]	0.5%
Forette	3	146	9	149	0.34	[0.09; 1.23]	1.2%
Harenberg − HESIM	11	810	8	780	1.32	[0.54; 3.27]	2.2%
Gardlund	48	5776	80	5917	0.61	[0.43; 0.88]	6.0%
Cohen − ARTEMIS	23	429	41	420	0.55	[0.34; 0.90]	4.6%
Goldhaber − ADOPT	22	2484	14	2488	1.57	[0.81; 3.07]	3.3%
Cohen − MAGELLAN	484	4050	373	4051	1.30	[1.14; 1.47]	8.3%
Cohen − APEX	190	3716	244	3716	0.78	[0.65; 0.94]	7.9%
Hull − EXCLAIM	25	2975	15	2988	1.67	[0.88; 3.17]	3.5%
Spyropoulos − MARINER	60	5982	55	6012	1.10	[0.76; 1.58]	5.9%
**Random effects model**		**34409**		**34588**	**0.77**	**[0.66; 0.90]**	**100.0%**

Heterogeneity: $I^2 = 72\%$, $\tau^2 = 0.07$, $p < 0.01$

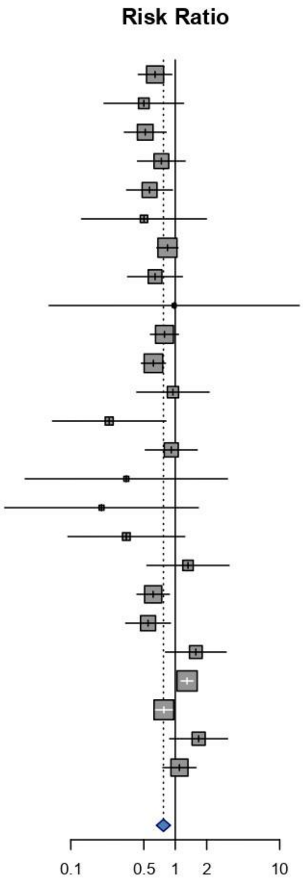

A.7. Major orthopedic and abdominal surgery, outcome: all-cause mortality

A relative risk (RR) > 1 mean that the experimental arm was associated with more mortality than the control arm

Study	Experimental Events	Experimental Total	Control Events	Control Total	RR	95%−CI	Weight
Levine	0.5	122	2.5	124	0.20	[0.01; 4.19]	0.4%
Leclerc	1.5	66	0.5	65	2.95	[0.12; 71.21]	0.3%
Colwell	1.0	400	2.0	210	0.26	[0.02; 2.88]	0.6%
Leyvraz A VALIDER	2.0	205	2.0	204	1.00	[0.14; 7.00]	0.9%
Haas	2.0	1013	2.0	1005	0.99	[0.14; 7.03]	0.9%
Colwell	9.0	1516	10.0	1495	0.89	[0.36; 2.18]	4.0%
Fitzgerald	1.0	173	3.0	176	0.34	[0.04; 3.23]	0.7%
Leclerc	2.0	336	2.0	334	0.99	[0.14; 7.02]	0.9%
Lassen − ADVANCE−1	3.0	1599	6.0	1596	0.50	[0.13; 1.99]	1.7%
Lassen − ADVANCE−2	3.0	1528	1.0	1529	3.00	[0.31; 28.83]	0.7%
Lassen − ADVANCE−3	5.0	2708	2.0	2699	2.49	[0.48; 12.83]	1.2%
Eriksson − RE−MODEL	2.0	1402	1.0	699	1.00	[0.09; 10.98]	0.6%
Eriksson − RE−NOVATE	6.5	2331	0.5	1162	6.48	[0.37; 114.94]	0.4%
Eriksson − RE−NOVATE−2	0.5	1036	2.5	1019	0.20	[0.01; 4.09]	0.4%
Ginsberg − RE−MOBILIZE	2.5	1739	0.5	876	2.52	[0.12; 52.41]	0.4%
Eriksson − RECORD 1	5.0	2266	4.0	2275	1.25	[0.34; 4.67]	1.9%
Lassen − RECORD 3	0.5	1254	6.5	1277	0.08	[0.00; 1.39]	0.4%
Turpie − RECORD 4	6.0	1584	6.0	1564	0.99	[0.32; 3.05]	2.6%
Bauer − PENTAMAKS	2.0	526	3.0	523	0.66	[0.11; 3.95]	1.0%
Eriksson − PENTHIFRA	40.0	849	43.0	862	0.94	[0.62; 1.44]	15.1%
Lassen − EPHESUS	2.0	1155	4.0	1154	0.50	[0.09; 2.72]	1.2%
Turpie − PENTATHLON	6.0	1138	3.0	1137	2.00	[0.50; 7.97]	1.7%
Heit	2.0	607	2.0	588	0.97	[0.14; 6.85]	0.9%
Comp	0.5	441	1.5	432	0.33	[0.01; 7.99]	0.3%
Eriksson − PENTHIFRA−Plus	6.0	326	8.0	330	0.76	[0.27; 2.16]	3.0%
Kakkar − RECORD2	2.0	1252	8.0	1257	0.25	[0.05; 1.18]	1.4%
Anderson	0.5	1717	1.5	1707	0.33	[0.01; 8.13]	0.3%
Lassen − SAVE−HIP1	2.0	1161	3.0	1165	0.67	[0.11; 4.00]	1.0%
Lassen − SAVE−HIP2	14.0	500	11.0	503	1.28	[0.59; 2.79]	5.2%
Lassen − SAVE−KNEE	0.5	576	2.5	574	0.20	[0.01; 4.14]	0.4%
Fisher − SAVE−HIP3	0.5	312	2.5	157	0.10	[0.00; 2.08]	0.4%
Ho	3.0	134	2.0	169	1.89	[0.32; 11.16]	1.1%
Rasmussen − FAME	20.0	205	17.0	222	1.27	[0.69; 2.36]	7.9%
Bergqvist − ENOXACAN II	3.0	253	6.0	248	0.49	[0.12; 1.94]	1.7%
Kakkar − CANBESURE	8.0	316	6.0	310	1.31	[0.46; 3.73]	3.0%
Turpie − APOLLO	8.0	650	5.0	659	1.62	[0.53; 4.93]	2.6%
Agnelli − PEGASUS	40.0	1465	55.0	1462	0.73	[0.49; 1.08]	16.2%
Kakkar − SAVE−ABDO	58.0	2175	43.0	2177	1.35	[0.91; 1.99]	16.9%
**Random effects model**		**37036**		**33945**	**0.97**	**[0.80; 1.16]**	**100.0%**

Heterogeneity: $I^2 = 0\%$, $\tau^2 = 0.01$, $p = 0.76$

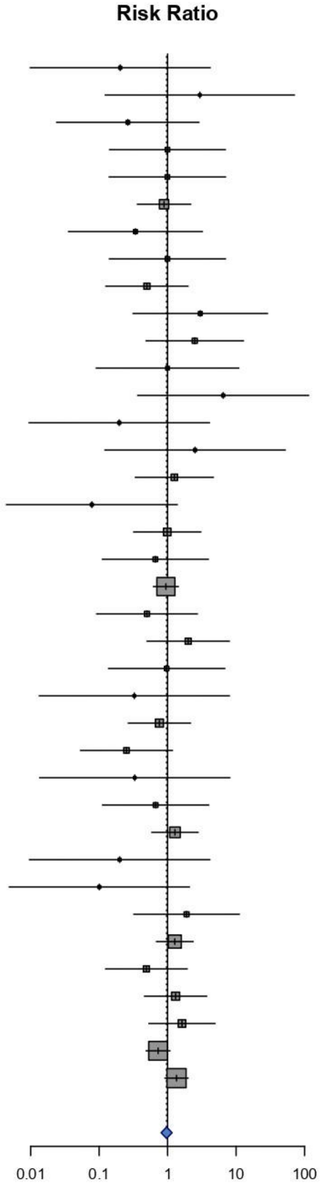

A.8. Major orthopedic and abdominal surgery, outcome: Net clinical benefit

A relative risk (RR) > 1 mean that the experimental arm was associated with more NCB than the control arm

Study	Experimental Events	Experimental Total	Control Events	Control Total	RR	95%−CI	Weight
Levine	32	122	64	124	0.51	[0.36; 0.72]	2.8%
Leclerc	13	66	38	65	0.34	[0.20; 0.57]	2.2%
Colwell	54	400	41	210	0.69	[0.48; 1.00]	2.7%
Leyvraz A VALIDER	24	205	40	204	0.60	[0.37; 0.95]	2.4%
Haas	212	1013	218	1005	0.96	[0.82; 1.14]	3.2%
Colwell	73	1516	64	1495	1.12	[0.81; 1.56]	2.8%
Fitzgerald	53	173	84	176	0.64	[0.49; 0.84]	3.0%
Leclerc	85	336	121	334	0.70	[0.55; 0.88]	3.1%
Lassen − ADVANCE−1	118	1599	126	1596	0.93	[0.73; 1.19]	3.1%
Lassen − ADVANCE−2	155	1528	257	1529	0.60	[0.50; 0.73]	3.2%
Lassen − ADVANCE−3	47	2708	91	2699	0.51	[0.36; 0.73]	2.8%
Eriksson − RE−MODEL	415	1402	202	699	1.02	[0.89; 1.18]	3.3%
Eriksson − RE−NOVATE	166	2331	78	1162	1.06	[0.82; 1.38]	3.0%
Eriksson − RE−NOVATE−2	77	1036	80	1019	0.95	[0.70; 1.28]	2.9%
Ginsberg − RE−MOBILIZE	417	1739	175	876	1.20	[1.03; 1.40]	3.3%
Eriksson − RECORD 1	22	2266	57	2275	0.39	[0.24; 0.63]	2.4%
Lassen − RECORD 3	91	1254	173	1277	0.54	[0.42; 0.68]	3.1%
Turpie − RECORD 4	79	1584	101	1564	0.77	[0.58; 1.03]	3.0%
Bauer − PENTAMAKS	58	526	105	523	0.55	[0.41; 0.74]	2.9%
Eriksson − PENTHIFRA	83	849	147	862	0.57	[0.45; 0.74]	3.1%
Lassen − EPHESUS	91	1155	123	1154	0.74	[0.57; 0.96]	3.0%
Turpie − PENTATHLON	87	1138	89	1137	0.98	[0.74; 1.30]	3.0%
Heit	9	607	13	588	0.67	[0.29; 1.56]	1.5%
Comp	59	441	96	432	0.60	[0.45; 0.81]	2.9%
Eriksson − PENTHIFRA−Plus	11	326	79	330	0.14	[0.08; 0.26]	2.0%
Kakkar − RECORD2	16	1252	76	1257	0.21	[0.12; 0.36]	2.2%
Anderson	11	1717	13	1707	0.84	[0.38; 1.87]	1.5%
Lassen − SAVE−HIP1	61	1161	117	1165	0.52	[0.39; 0.71]	2.9%
Lassen − SAVE−HIP2	69	500	81	503	0.86	[0.64; 1.15]	2.9%
Lassen − SAVE−KNEE	108	576	124	574	0.87	[0.69; 1.09]	3.1%
Fisher − SAVE−HIP3	10	312	19	157	0.26	[0.13; 0.56]	1.7%
Ho	3	134	6	169	0.63	[0.16; 2.47]	0.7%
Rasmussen − FAME	13	205	33	222	0.43	[0.23; 0.79]	2.0%
Bergqvist − ENOXACAN II	10	253	24	248	0.41	[0.20; 0.84]	1.7%
Kakkar − CANBESURE	23	316	33	310	0.68	[0.41; 1.14]	2.3%
Turpie − APOLLO	17	650	23	659	0.75	[0.40; 1.39]	2.0%
Agnelli − PEGASUS	102	1465	105	1462	0.97	[0.75; 1.26]	3.0%
Kakkar − SAVE−ABDO	149	2175	175	2177	0.85	[0.69; 1.05]	3.2%
**Random effects model**		**37036**		**33945**	**0.66**	**[0.58; 0.76]**	**100.0%**

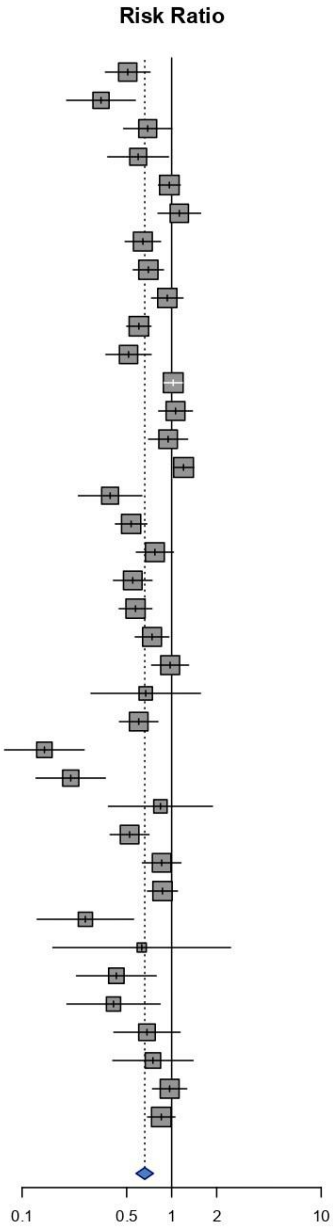

Heterogeneity: I^2 = 82%, τ^2 = 0.14, p < 0.01

A.9. Thromboprophylaxis in cancer patients, outcome: All-caus mortality

A relative risk (RR) > 1 mean that the experimental arm was associated with more mortality than the control arm

Study	Experimental Events	Experimental Total	Control Events	Control Total	RR	95%-CI	Weight
Haas – TOPIC-1	15	174	12	178	1.28	[0.62; 2.65]	0.7%
Haas – TOPIC-2	55	273	59	273	0.93	[0.67; 1.29]	3.4%
Perry – PRODIGE	18	99	11	87	1.44	[0.72; 2.87]	0.8%
Maraveyas – FRAGEM	7	63	4	60	1.67	[0.51; 5.40]	0.3%
Klerk – MALT	6	148	15	154	0.42	[0.17; 1.04]	0.4%
Agnelli – PROTECHT	333	769	155	381	1.06	[0.92; 1.23]	17.2%
van Doormaal – INPACT	138	244	160	259	0.92	[0.79; 1.06]	17.2%
Agnelli – SAVE-ONCO	698	1608	714	1604	0.98	[0.90; 1.05]	60.0%
**Random effects model**		**3378**		**2996**	**0.99**	**[0.92; 1.06]**	**100.0%**

Heterogeneity: $I^2 = 12\%$, $\tau^2 < 0.01$, $p = 0.33$

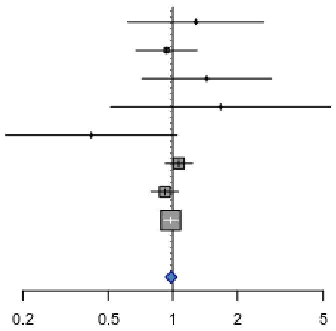

A.10. Thromboprophylaxis in cancer patients, outcome: Net clinical benefit

A relative risk (RR) > 1 mean that the experimental arm was associated with more NCB than the control arm

Study	Experimental Events	Experimental Total	Control Events	Control Total	RR	95%-CI	Weight
Haas – TOPIC-1	10	174	7	178	1.46	[0.57; 3.75]	9.4%
Haas – TOPIC-2	22	273	28	273	0.79	[0.46; 1.34]	15.3%
Perry – PRODIGE	12	99	13	87	0.81	[0.39; 1.68]	12.2%
Maraveyas – FRAGEM	16	62	4	59	3.81	[1.35; 10.73]	8.4%
Klerk – MALT	7	148	4	154	1.82	[0.54; 6.09]	6.9%
Agnelli – PROTECHT	26	769	19	381	0.68	[0.38; 1.21]	14.5%
van Doormaal – INPACT	26	244	24	259	1.15	[0.68; 1.95]	15.4%
Agnelli – SAVE-ONCO	39	1608	73	1604	0.53	[0.36; 0.78]	17.8%
**Random effects model**		**3377**		**2995**	**0.95**	**[0.67; 1.35]**	**100.0%**

Heterogeneity: $I^2 = 62\%$, $\tau^2 = 0.18$, $p = 0.01$

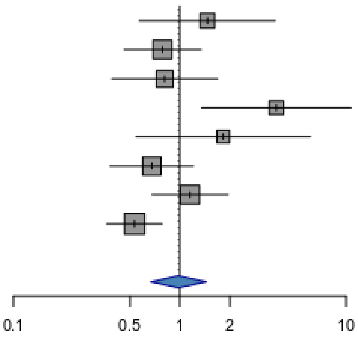

Supplement: Supplementary file 1 — Supplementary Information 1. [file 41598_2021_94160_MOESM1_ESM.pdf]
