# Supplementary material for: Meta-regression of randomized control trials with antithrombotics: weak correlation between net clinical benefit and all cause-mortality
Source: Sci Rep. 2021 Jul 19;11:14728. doi: 10.1038/s41598-021-94160-1 (PMC8290002; doi:10.1038/s41598-021-94160-1)

Appendix C - Forest plot of relative risks (RR) for all-cause mortality and net clinical benefit (NCB) for antiplatelet, vitamin k antagonist (VKA), direct oral anticoagulants (DOAC), and Low-molecular-weight heparin (LMWH)

C.1. Antiplatelet, outcome: All-cause mortality

A relative risk (RR) > 1 mean that the experimental arm was associated with more mortality than the control arm

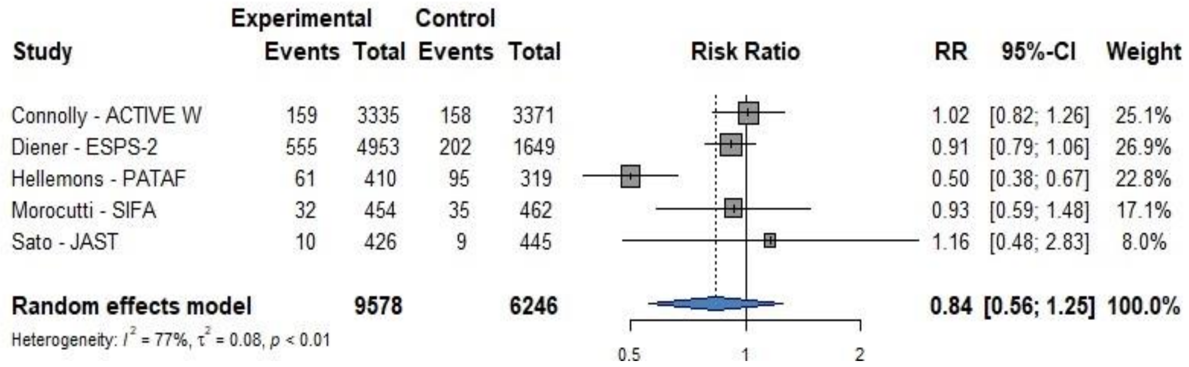

C.2. Antiplatelet, outcome: NCB

A relative risk (RR) > 1 mean that the experimental arm was associated with more mortality than the control arm

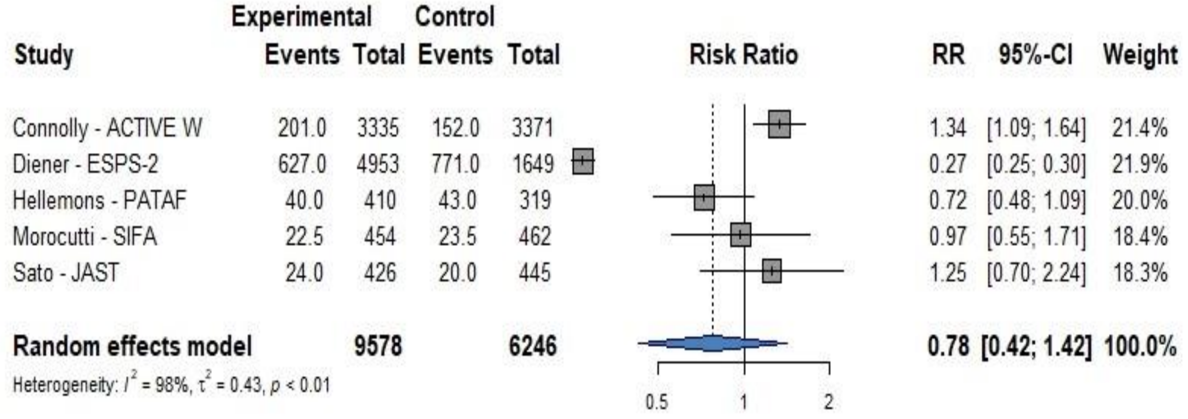

C.3. VKA, outcome: All-cause mortality

A relative risk (RR) > 1 mean that the experimental arm was associated with more mortality than the control arm

Study	Experimental Events	Experimental Total	Control Events	Control Total	RR	95%-CI	Weight
Koudstaal - EAFT	143	629	143	378	0.60	[0.50; 0.73]	12.6%
Connolly - CAFA	10	187	8	191	1.28	[0.52; 3.16]	4.9%
Ezekowitz - SPINAF	15	260	22	265	0.69	[0.37; 1.31]	7.3%
Gullov - AFASAK II	25	507	17	170	0.49	[0.27; 0.89]	7.8%
Kistler - BAATAF	11	212	26	208	0.42	[0.21; 0.82]	6.8%
Mant - BAFTA	107	488	108	485	0.98	[0.78; 1.25]	12.2%
McBride - SPAF I	45	762	58	568	0.58	[0.40; 0.84]	10.5%
McBride - SPAF III	42	521	35	523	1.20	[0.78; 1.86]	9.7%
Pengo	78	135	70	132	1.09	[0.88; 1.35]	12.4%
Pérez-Gomez - NASPEAFa	26	479	35	235	0.36	[0.22; 0.59]	9.1%
Pérez-Gomez - NASPEAFb	12	236	23	259	0.57	[0.29; 1.12]	6.9%
**Random effects model**		**4416**		**3414**	**0.70**	**[0.53; 0.93]**	**100.0%**

Heterogeneity: $I^2 = 77\%$, $\tau^2 = 0.12$, $p < 0.01$

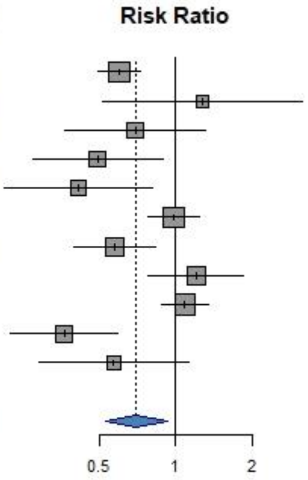

C.4. VKA, outcome: NCB

A relative risk (RR) > 1 mean that the experimental arm was associated with more mortality than the control arm

Study	Experimental Events	Experimental Total	Control Events	Control Total	RR	95%-CI	Weight
Koudstaal - EAFT	127	629	147	378	0.52	[0.42; 0.63]	11.1%
Connolly - CAFA	6	187	3	191	2.04	[0.52; 8.05]	4.8%
Ezekowitz - SPINAF	10	260	29	265	0.35	[0.17; 0.71]	8.4%
Gullov - AFASAK II	38	507	42	170	0.30	[0.20; 0.45]	10.2%
Kistler - BAATAF	4	212	14	208	0.28	[0.09; 0.84]	6.1%
Mant - BAFTA	46	488	69	485	0.66	[0.47; 0.94]	10.5%
McBride - SPAF I	43	762	77	568	0.42	[0.29; 0.59]	10.4%
McBride - SPAF III	56	521	23	523	2.44	[1.53; 3.91]	9.8%
Pengo	23	135	35	132	0.64	[0.40; 1.03]	9.8%
Pérez-Gomez - NASPEAFa	24	479	29	235	0.41	[0.24; 0.68]	9.5%
Pérez-Gomez - NASPEAFb	22	236	25	259	0.97	[0.56; 1.67]	9.4%
**Random effects model**		**4416**		**3414**	**0.60**	**[0.41; 0.90]**	**100.0%**

Heterogeneity: $I^2 = 84\%$, $\tau^2 = 0.36$, $p < 0.01$

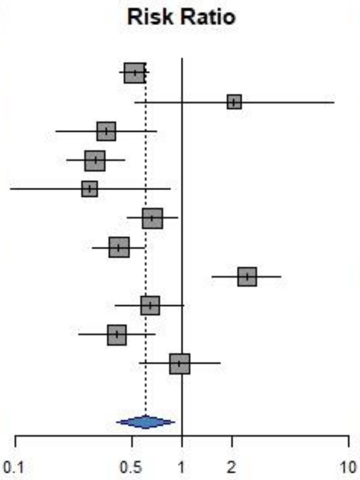

C.5. DOAC, outcome: All-cause mortality

A relative risk (RR) > 1 mean that the experimental arm was associated with more mortality than the control arm

Study	Experimental Events	Experimental Total	Control Events	Control Total	RR	95%−CI	Weight
BÜller − AMADEUS	62.0	2283	61.0	2293	1.02	[0.72; 1.45]	3.5%
Connolly − AVERROES	111.0	2808	140.0	2791	0.79	[0.62; 1.01]	4.4%
Connolly − RE−LY	884.0	12091	974.0	6022	0.45	[0.41; 0.49]	5.6%
Giugliano − ENGAGE AF−TIMI	1510.0	14069	839.0	7036	0.90	[0.83; 0.97]	5.6%
Granger − ARISTOTLE	603.0	9120	669.0	9081	0.90	[0.81; 1.00]	5.5%
Hori − J−ROCKET AF	7.0	640	5.0	640	1.40	[0.45; 4.39]	0.7%
Patel − ROCKET AF	208.0	7061	250.0	7082	0.83	[0.70; 1.00]	4.9%
Connolly − EXPLORE−Xa	1.0	381	1.0	127	0.33	[0.02; 5.29]	0.1%
Büller − CASSIOPEA	89.0	1599	84.0	1603	1.06	[0.79; 1.42]	4.0%
Büller − COLOMBUS	36.0	501	39.0	511	0.94	[0.61; 1.46]	2.9%
Büller − EINSTEIN DVT	38.0	1731	49.0	1718	0.77	[0.51; 1.17]	3.0%
Büller − EINSTEIN DVT dose ranging	4.0	136	5.0	137	0.81	[0.22; 2.94]	0.6%
Büller − EINSTEIN PE	58.0	2420	50.0	2413	1.16	[0.80; 1.68]	3.3%
Büller − EQUINOX	11.0	386	18.0	371	0.59	[0.28; 1.23]	1.5%
Büller − HOKUSAI VTE	132.0	4143	126.0	4149	1.05	[0.83; 1.33]	4.4%
Büller − VAN GOGH DVT	55.0	1452	44.0	1452	1.25	[0.85; 1.85]	3.2%
Büller − VAN GOGH PE	64.0	1095	45.0	1120	1.45	[1.00; 2.11]	3.3%
Agnelli − AMPLIFY	41.0	2691	52.0	2704	0.79	[0.53; 1.19]	3.1%
Agnelli − AMPLIFY EXT	11.0	1657	14.0	829	0.39	[0.18; 0.86]	1.3%
Schulman − RE−COVER	21.0	1274	21.0	1265	0.99	[0.55; 1.81]	2.0%
Schulman − RE−COVER II	29.0	1279	26.0	1289	1.12	[0.67; 1.90]	2.3%
Schulman − RE−MEDY	17.0	1435	19.0	1431	0.89	[0.47; 1.71]	1.8%
Büller − VAN GOGH EXT	9.0	594	4.0	621	2.35	[0.73; 7.60]	0.7%
Cohen − ARTEMIS	14.0	429	25.0	420	0.55	[0.29; 1.04]	1.8%
Goldhaber − ADOPT	131.0	2484	133.0	2488	0.99	[0.78; 1.25]	4.5%
Cohen − MAGELLAN	231.0	4050	224.0	4051	1.03	[0.86; 1.23]	4.9%
Cohen − APEX	210.0	3716	215.0	3716	0.98	[0.81; 1.17]	4.9%
Spyropoulos − MARINER	71.0	6007	89.0	6012	0.80	[0.59; 1.09]	3.8%
Lassen − ADVANCE−1	3.0	1599	6.0	1596	0.50	[0.13; 1.99]	0.5%
Lassen − ADVANCE−2	3.0	1528	1.0	1529	3.00	[0.31; 28.83]	0.2%
Lassen − ADVANCE−3	5.0	2708	2.0	2699	2.49	[0.48; 12.83]	0.4%
Eriksson − RE−MODEL	2.0	1402	1.0	699	1.00	[0.09; 10.98]	0.2%
Eriksson − RE−NOVATE	6.5	2331	0.5	1162	6.48	[0.37; 114.94]	0.1%
Eriksson − RE−NOVATE−2	0.5	1036	2.5	1019	0.20	[0.01; 4.09]	0.1%
Ginsberg − RE−MOBILIZE	2.5	1739	0.5	876	2.52	[0.12; 52.41]	0.1%
Eriksson − RECORD 1	5.0	2266	4.0	2275	1.25	[0.34; 4.67]	0.6%
Lassen − RECORD 3	0.5	1254	6.5	1277	0.08	[0.00; 1.39]	0.1%
Turpie − RECORD 4	6.0	1584	6.0	1564	0.99	[0.32; 3.05]	0.7%
Bauer − PENTAMAKS	2.0	526	3.0	523	0.66	[0.11; 3.95]	0.3%
Eriksson − PENTHIFRA	40.0	849	43.0	862	0.94	[0.62; 1.44]	3.0%
Lassen − EPHESUS	2.0	1155	4.0	1154	0.50	[0.09; 2.72]	0.4%
Turpie − PENTATHLON	6.0	1138	3.0	1137	2.00	[0.50; 7.97]	0.5%
Eriksson − PENTHIFRA−Plus	6.0	326	8.0	330	0.76	[0.27; 2.16]	0.8%
Kakkar − RECORD2	2.0	1252	8.0	1257	0.25	[0.05; 1.18]	0.4%
Anderson	0.5	1717	1.5	1707	0.33	[0.01; 8.13]	0.1%
Turpie − APOLLO	8.0	650	5.0	659	1.62	[0.53; 4.93]	0.8%
Agnelli − PEGASUS	40.0	1465	55.0	1462	0.73	[0.49; 1.08]	3.1%
**Random effects model**		**114057**		**97159**	**0.89**	**[0.81; 0.99]**	**100.0%**

Heterogeneity: $I^2 = 82\%$, $\tau^2 = 0.05$, $p < 0.01$

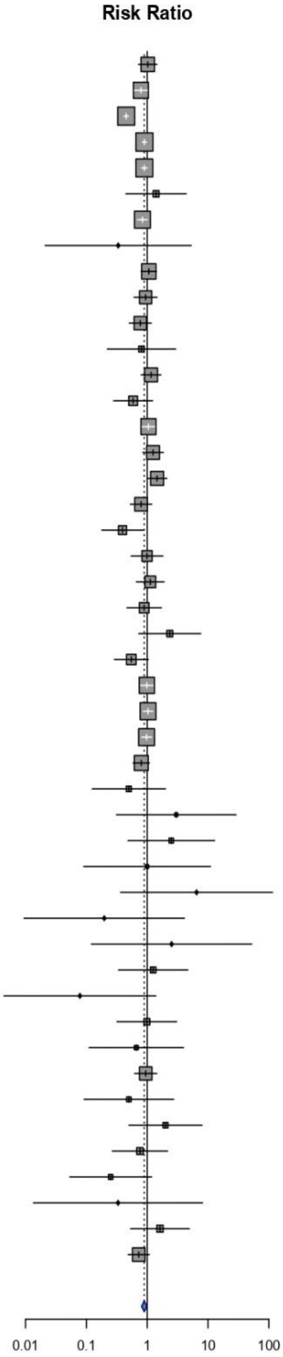

C.6. DOAC, outcome: NCB

A relative risk (RR) > 1 mean that the experimental arm was associated with more mortality than the control arm

Study	Experimental Events	Experimental Total	Control Events	Control Total	RR	95%-CI	Weight
BÜller − AMADEUS	92.0	2283	56.0	2293	1.65	[1.19; 2.29]	2.2%
Connolly − AVERROES	93.0	2808	144.0	2791	0.64	[0.50; 0.83]	2.4%
Connolly − RE−LY	1014.0	12091	1212.0	6022	0.42	[0.39; 0.45]	2.6%
Giugliano − ENGAGE AF−TIMI	1313.0	14069	1682.0	7036	0.39	[0.37; 0.42]	2.6%
Granger − ARISTOTLE	526.0	9120	712.0	9081	0.74	[0.66; 0.82]	2.5%
Hori − J−ROCKET AF	37.0	640	57.0	640	0.65	[0.44; 0.97]	2.1%
Patel − ROCKET AF	579.0	7111	607.0	7125	0.96	[0.86; 1.07]	2.5%
Connolly − EXPLORE−Xa	5.5	381	5.5	127	0.33	[0.10; 1.07]	0.9%
Büller − CASSIOPEA	68.0	1599	136.0	1603	0.50	[0.38; 0.67]	2.3%
Büller − COLOMBUS	37.0	501	33.0	511	1.14	[0.73; 1.80]	2.0%
Büller − EINSTEIN DVT	50.0	1731	71.0	1718	0.70	[0.49; 1.00]	2.2%
Büller − EINSTEIN DVT dose ranging	4.0	136	9.0	137	0.45	[0.14; 1.42]	0.9%
Büller − EINSTEIN PE	76.0	2420	96.0	2413	0.79	[0.59; 1.06]	2.3%
Büller − EQUINOX	13.0	386	30.0	371	0.42	[0.22; 0.79]	1.6%
Büller − HOKUSAI VTE	186.0	4143	212.0	4149	0.88	[0.73; 1.06]	2.4%
Büller − VAN GOGH DVT	54.0	1452	59.0	1452	0.92	[0.64; 1.31]	2.2%
Büller − VAN GOGH PE	52.0	1095	44.0	1120	1.21	[0.82; 1.79]	2.1%
Agnelli − AMPLIFY	74.0	2691	120.0	2704	0.62	[0.47; 0.82]	2.3%
Agnelli − AMPLIFY EXT	39.0	1657	79.0	829	0.25	[0.17; 0.36]	2.1%
Schulman − RE−COVER	54.0	1274	56.0	1265	0.96	[0.66; 1.38]	2.2%
Schulman − RE−COVER II	49.0	1279	52.0	1289	0.95	[0.65; 1.39]	2.1%
Schulman − RE−MEDY	39.0	1435	43.0	1431	0.90	[0.59; 1.39]	2.0%
Büller − VAN GOGH EXT	17.0	594	23.0	621	0.77	[0.42; 1.43]	1.7%
Cohen − ARTEMIS	23.0	429	41.0	420	0.55	[0.34; 0.90]	1.9%
Goldhaber − ADOPT	22.0	2484	14.0	2488	1.57	[0.81; 3.07]	1.6%
Cohen − MAGELLAN	484.0	4050	373.0	4051	1.30	[1.14; 1.47]	2.5%
Cohen − APEX	190.0	3716	244.0	3716	0.78	[0.65; 0.94]	2.5%
Spyropoulos − MARINER	60.0	5982	55.0	6012	1.10	[0.76; 1.58]	2.2%
Lassen − ADVANCE−1	118.0	1599	126.0	1596	0.93	[0.73; 1.19]	2.4%
Lassen − ADVANCE−2	155.0	1528	257.0	1529	0.60	[0.50; 0.73]	2.5%
Lassen − ADVANCE−3	47.0	2708	91.0	2699	0.51	[0.36; 0.73]	2.2%
Eriksson − RE−MODEL	415.0	1402	202.0	699	1.02	[0.89; 1.18]	2.5%
Eriksson − RE−NOVATE	166.0	2331	78.0	1162	1.06	[0.82; 1.38]	2.3%
Eriksson − RE−NOVATE−2	77.0	1036	80.0	1019	0.95	[0.70; 1.28]	2.3%
Ginsberg − RE−MOBILIZE	417.0	1739	175.0	876	1.20	[1.03; 1.40]	2.5%
Eriksson − RECORD 1	22.0	2266	57.0	2275	0.39	[0.24; 0.63]	1.9%
Lassen − RECORD 3	91.0	1254	173.0	1277	0.54	[0.42; 0.68]	2.4%
Turpie − RECORD 4	79.0	1584	101.0	1564	0.77	[0.58; 1.03]	2.3%
Bauer − PENTAMAKS	58.0	526	105.0	523	0.55	[0.41; 0.74]	2.3%
Eriksson − PENTHIFRA	83.0	849	147.0	862	0.57	[0.45; 0.74]	2.4%
Lassen − EPHESUS	91.0	1155	123.0	1154	0.74	[0.57; 0.96]	2.3%
Turpie − PENTATHLON	87.0	1138	89.0	1137	0.98	[0.74; 1.30]	2.3%
Eriksson − PENTHIFRA−Plus	11.0	326	79.0	330	0.14	[0.08; 0.26]	1.7%
Kakkar − RECORD2	16.0	1252	76.0	1257	0.21	[0.12; 0.36]	1.8%
Anderson	11.0	1717	13.0	1707	0.84	[0.38; 1.87]	1.3%
Turpie − APOLLO	17.0	650	23.0	659	0.75	[0.40; 1.39]	1.7%
Agnelli − PEGASUS	102.0	1465	105.0	1462	0.97	[0.75; 1.26]	2.3%
**Random effects model**		**114082**		**97202**	**0.71**	**[0.62; 0.81]**	**100.0%**

Heterogeneity: $I^2 = 95\%$, $\tau^2 = 0.18$, $p < 0.01$

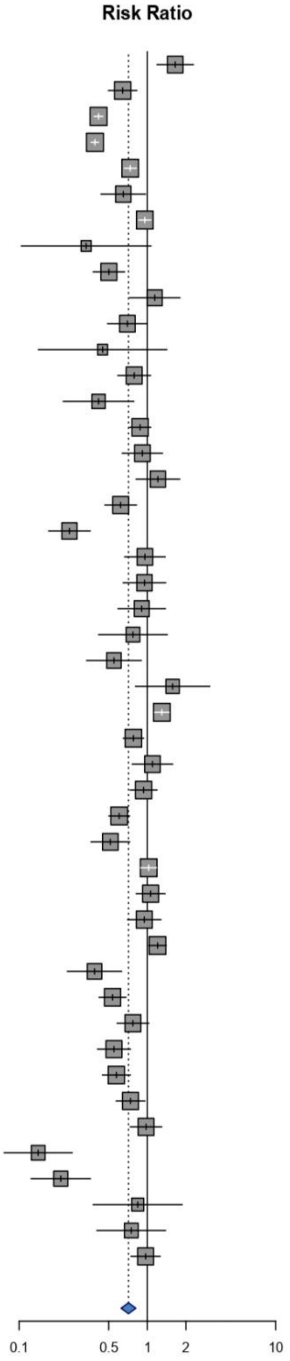

C.7. LMWH, outcome: All-cause mortalityA relative risk (RR) > 1 mean that the experimental arm was associated with more mortality than the control arm

Study	Experimental Events	Experimental Total	Control Events	Control Total	RR	95%-CI	Weight
Büller - COLOMBUS	36.0	501	39.0	511	0.94	[0.61; 1.46]	1.1%
Büller - MATISSE DVT	41.0	1098	33.0	1107	1.25	[0.80; 1.97]	1.0%
Büller - MATISSE PE	57.0	1103	48.0	1110	1.20	[0.82; 1.74]	1.5%
Brandjes	1.0	60	2.0	60	0.50	[0.05; 5.37]	0.0%
Hull	10.0	213	21.0	219	0.49	[0.24; 1.01]	0.4%
Kearon - FIDO	22.0	353	18.0	355	1.23	[0.67; 2.25]	0.6%
Levine	11.0	247	17.0	253	0.66	[0.32; 1.39]	0.4%
Merli	18.0	610	9.0	290	0.95	[0.43; 2.09]	0.3%
Prandoni - GALILEI	12.0	360	12.0	360	1.00	[0.46; 2.20]	0.3%
Prandoni et al	6.0	85	12.0	85	0.50	[0.20; 1.27]	0.2%
Simonneau - THÉSÉE	12.0	304	14.0	308	0.87	[0.41; 1.85]	0.4%
Eriksson - THRIVE I	1.5	68	0.5	73	3.22	[0.13; 77.72]	0.0%
Leizorovicz - PREVENT	107.0	1856	103.0	1850	1.04	[0.80; 1.35]	3.1%
Lederle	36.0	140	32.0	140	1.12	[0.74; 1.70]	1.2%
Samama - MEDENOX	41.0	367	50.0	371	0.83	[0.56; 1.22]	1.4%
Fraisse	8.0	109	8.0	114	1.05	[0.41; 2.69]	0.2%
Mahe	124.0	1230	128.0	1244	0.98	[0.78; 1.24]	3.9%
Diener - PROTECT	21.0	272	15.0	273	1.41	[0.74; 2.67]	0.5%
Riess - CERTIFY	66.0	1626	72.0	1618	0.91	[0.66; 1.26]	2.0%
Schellong - CERTAIN	9.0	163	15.0	174	0.64	[0.29; 1.42]	0.3%
Harenberg	3.0	84	1.0	82	2.93	[0.31; 27.58]	0.0%
Hillbom	21.0	106	28.0	106	0.75	[0.46; 1.23]	0.9%
Sherman - PREVAIL	100.0	884	103.0	878	0.96	[0.74; 1.25]	3.2%
Bergmann - EMSG	7.0	217	8.0	225	0.91	[0.33; 2.46]	0.2%
Lechler - PRIME	7.0	477	11.0	482	0.64	[0.25; 1.64]	0.2%
Kleber - PRINCE	9.0	332	15.0	333	0.60	[0.27; 1.36]	0.3%
Aquino	1.0	49	2.0	50	0.51	[0.05; 5.45]	0.0%
Manciet - APTE	8.0	129	8.0	127	0.98	[0.38; 2.54]	0.2%
Forette	6.0	146	7.0	149	0.87	[0.30; 2.54]	0.2%
Harenberg - HESIM	23.0	810	9.0	780	2.46	[1.15; 5.28]	0.4%
Gardlund	304.0	5776	333.0	5917	0.94	[0.80; 1.09]	9.3%
Hull - EXCLAIM	148.0	2975	142.0	2988	1.05	[0.84; 1.31]	4.2%
Levine	0.5	122	2.5	124	0.20	[0.01; 4.19]	0.0%
Leclerc	1.5	66	0.5	65	2.95	[0.12; 71.21]	0.0%
Colwell	1.0	400	2.0	210	0.26	[0.02; 2.88]	0.0%
Leyvraz A VALIDER	2.0	205	2.0	204	1.00	[0.14; 7.00]	0.1%
Haas	2.0	1013	2.0	1005	0.99	[0.14; 7.03]	0.1%
Colwell	9.0	1516	10.0	1495	0.89	[0.36; 2.18]	0.3%
Fitzgerald	1.0	173	3.0	176	0.34	[0.04; 3.23]	0.0%
Leclerc	2.0	336	2.0	334	0.99	[0.14; 7.02]	0.1%
Heit	2.0	607	2.0	588	0.97	[0.14; 6.85]	0.1%
Comp	0.5	441	1.5	432	0.33	[0.01; 7.99]	0.0%
Lassen - SAVE-HIP1	2.0	1161	3.0	1165	0.67	[0.11; 4.00]	0.1%
Lassen - SAVE-HIP2	14.0	500	11.0	503	1.28	[0.59; 2.79]	0.4%
Lassen - SAVE-KNEE	0.5	576	2.5	574	0.20	[0.01; 4.14]	0.0%
Fisher - SAVE-HIP3	0.5	312	2.5	157	0.10	[0.00; 2.08]	0.0%
Ho	3.0	134	2.0	169	1.89	[0.32; 11.16]	0.1%
Rasmussen - FAME	20.0	205	17.0	222	1.27	[0.69; 2.36]	0.6%
Bergqvist - ENOXACAN II	3.0	253	6.0	248	0.49	[0.12; 1.94]	0.1%
Kakkar - CANBESURE	8.0	316	6.0	310	1.31	[0.46; 3.73]	0.2%
Kakkar - SAVE-ABDO	58.0	2175	43.0	2177	1.35	[0.91; 1.99]	1.4%
Haas - TOPIC-1	15.0	174	12.0	178	1.28	[0.62; 2.65]	0.4%
Haas - TOPIC-2	55.0	273	59.0	273	0.93	[0.67; 1.29]	2.0%
Perry - PRODIGE	18.0	99	11.0	87	1.44	[0.72; 2.87]	0.4%
Maraveyas - FRAGEM	7.0	63	4.0	60	1.67	[0.51; 5.40]	0.2%
Klerk - MALT	6.0	148	15.0	154	0.42	[0.17; 1.04]	0.3%
Agnelli - PROTECHT	333.0	769	155.0	381	1.06	[0.92; 1.23]	10.0%
van Doormaal - INPACT	138.0	244	160.0	259	0.92	[0.79; 1.06]	10.0%
Agnelli - SAVE-ONCO	698.0	1608	714.0	1604	0.98	[0.90; 1.05]	34.9%
**Random effects model**		**36639**		**35786**	**0.98**	**[0.94; 1.02]**	**100.0%**

Heterogeneity: $I^2 = 0\%$, $\tau^2 = 0$, $p = 0.88$

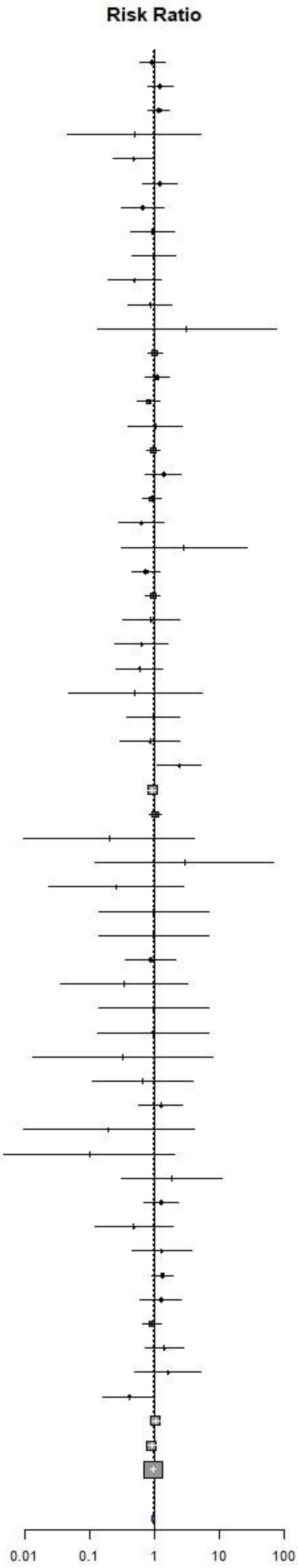

C.8. LMWH, outcome: NCB

A relative risk (RR) > 1 mean that the experimental arm was associated with more mortality than the control arm

Study	Experimental Events	Experimental Total	Control Events	Control Total	RR	95%-CI	Weight
Büller - COLOMBUS	37	501	33	511	1.14	[0.73; 1.80]	2.0%
Büller - MATISSE DVT	71	1098	71	1107	1.01	[0.73; 1.39]	2.8%
Büller - MATISSE PE	64	1103	82	1110	0.79	[0.57; 1.08]	2.8%
Brandjes	6	60	15	60	0.40	[0.17; 0.96]	0.8%
Hull	7	213	26	219	0.28	[0.12; 0.62]	0.9%
Kearon - FIDO	17	353	17	355	1.01	[0.52; 1.94]	1.3%
Levine	18	247	20	253	0.92	[0.50; 1.70]	1.4%
Merli	31	610	18	290	0.82	[0.47; 1.44]	1.6%
Prandoni - GALILEI	17	360	19	360	0.89	[0.47; 1.69]	1.3%
Prandoni et al	7	85	15	85	0.47	[0.20; 1.09]	0.9%
Simonneau - THÉSÉE	8	304	11	308	0.74	[0.30; 1.81]	0.8%
Eriksson - THRIVE I	3	68	6	73	0.54	[0.14; 2.06]	0.4%
Leizorovicz - PREVENT	45	1856	70	1850	0.64	[0.44; 0.93]	2.5%
Lederle	7	140	14	140	0.50	[0.21; 1.20]	0.8%
Samama - MEDENOX	25	367	49	371	0.52	[0.33; 0.82]	2.0%
Fraisse	19	109	27	114	0.74	[0.44; 1.24]	1.7%
Mahe	23	1230	41	1244	0.57	[0.34; 0.94]	1.8%
Diener - PROTECT	3	272	6	273	0.50	[0.13; 1.99]	0.4%
Riess - CERTIFY	121	1626	143	1618	0.84	[0.67; 1.06]	3.4%
Schellong - CERTAIN	15	163	25	174	0.64	[0.35; 1.17]	1.4%
Harenberg	1	84	1	82	0.98	[0.06; 15.35]	0.1%
Hillbom	41	106	52	106	0.79	[0.58; 1.07]	2.9%
Sherman - PREVAIL	79	884	127	878	0.62	[0.47; 0.81]	3.2%
Bergmann - EMSG	11	217	12	225	0.95	[0.43; 2.11]	1.0%
Lechler - PRIME	3	477	13	482	0.23	[0.07; 0.81]	0.4%
Kleber - PRINCE	21	332	23	333	0.92	[0.52; 1.62]	1.5%
Aquino	1	49	3	50	0.34	[0.04; 3.16]	0.2%
Manciet - APTE	1	129	5	127	0.20	[0.02; 1.66]	0.2%
Forette	3	146	9	149	0.34	[0.09; 1.23]	0.4%
Harenberg - HESIM	11	810	8	780	1.32	[0.54; 3.27]	0.8%
Gardlund	48	5776	80	5917	0.61	[0.43; 0.88]	2.6%
Hull - EXCLAIM	25	2975	15	2988	1.67	[0.88; 3.17]	1.3%
Levine	32	122	64	124	0.51	[0.36; 0.72]	2.6%
Leclerc	13	66	38	65	0.34	[0.20; 0.57]	1.7%
Colwell	54	400	41	210	0.69	[0.48; 1.00]	2.5%
Leyvraz A VALIDER	24	205	40	204	0.60	[0.37; 0.95]	2.0%
Haas	212	1013	218	1005	0.96	[0.82; 1.14]	3.8%
Colwell	73	1516	64	1495	1.12	[0.81; 1.56]	2.7%
Fitzgerald	53	173	84	176	0.64	[0.49; 0.84]	3.1%
Leclerc	85	336	121	334	0.70	[0.55; 0.88]	3.4%
Heit	9	607	13	588	0.67	[0.29; 1.56]	0.9%
Comp	59	441	96	432	0.60	[0.45; 0.81]	2.9%
Lassen - SAVE-HIP1	61	1161	117	1165	0.52	[0.39; 0.71]	2.9%
Lassen - SAVE-HIP2	69	500	81	503	0.86	[0.64; 1.15]	2.9%
Lassen - SAVE-KNEE	108	576	124	574	0.87	[0.69; 1.09]	3.4%
Fisher - SAVE-HIP3	10	312	19	157	0.26	[0.13; 0.56]	1.1%
Ho	3	134	6	169	0.63	[0.16; 2.47]	0.4%
Rasmussen - FAME	13	205	33	222	0.43	[0.23; 0.79]	1.4%
Bergqvist - ENOXACAN II	10	253	24	248	0.41	[0.20; 0.84]	1.1%
Kakkar - CANBESURE	23	316	33	310	0.68	[0.41; 1.14]	1.8%
Kakkar - SAVE-ABDO	149	2175	175	2177	0.85	[0.69; 1.05]	3.5%
Haas - TOPIC-1	10	174	7	178	1.46	[0.57; 3.75]	0.7%
Haas - TOPIC-2	22	273	28	273	0.79	[0.46; 1.34]	1.7%
Perry - PRODIGE	12	99	13	87	0.81	[0.39; 1.68]	1.1%
Maraveyas - FRAGEM	16	62	4	59	3.81	[1.35; 10.73]	0.6%
Klerk - MALT	7	148	4	154	1.82	[0.54; 6.09]	0.5%
Agnelli - PROTECHT	26	769	19	381	0.68	[0.38; 1.21]	1.5%
van Doormaal - INPACT	26	244	24	259	1.15	[0.68; 1.95]	1.7%
Agnelli - SAVE-ONCO	39	1608	73	1604	0.53	[0.36; 0.78]	2.4%
**Random effects model**		**36638**		**35785**	**0.72**	**[0.66; 0.78]**	**100.0%**

Heterogeneity: $I^2 = 51\%$, $\tau^2 = 0.05$, $p < 0.01$

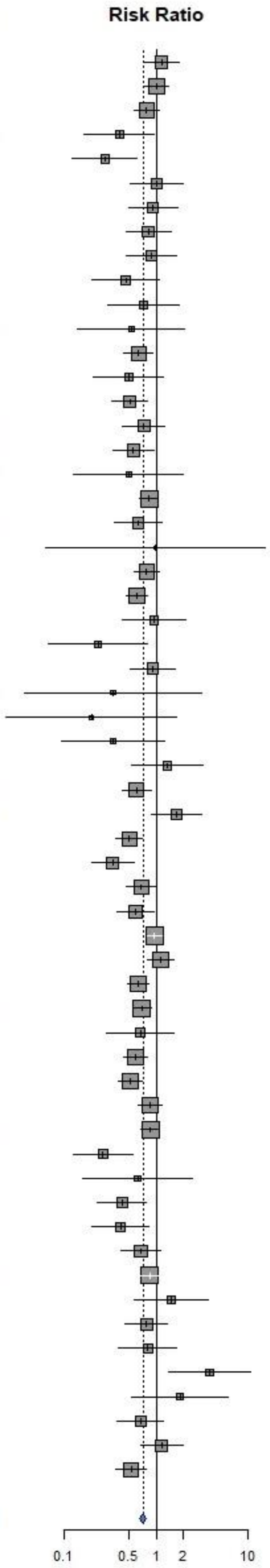

Supplement: Supplementary file 2 — Supplementary Information 2. [file 41598_2021_94160_MOESM2_ESM.pdf]
