# Supplementary material for: Meta-regression of randomized control trials with antithrombotics: weak correlation between net clinical benefit and all cause-mortality
Source: Sci Rep. 2021 Jul 19;11:14728. doi: 10.1038/s41598-021-94160-1 (PMC8290002; doi:10.1038/s41598-021-94160-1)
Supplement: Supplementary file 3 — Supplementary Information 3. [file 41598_2021_94160_MOESM3_ESM.pdf]

APPENDIX C: STUDIES WITH FULL REFERENCES

C.1 NON-VALVULAR ATRIAL FIBRILLATION

Author, year	Study acronym	Full reference
Koudstaal, 1993	EAFT	(1) Secondary prevention in non-rheumatic atrial fibrillation after transient ischaemic attack or minor stroke. EAFT (European Atrial Fibrillation Trial) Study Group. *Lancet Lond. Engl.* **342**, 1255–1262 (1993).
BÜller, 2008	AMADEUS	(2) Amadeus Investigators *et al.* Comparison of idraparinux with vitamin K antagonists for prevention of thromboembolism in patients with atrial fibrillation: a randomised, open-label, non-inferiority trial. *Lancet Lond. Engl.* **371**, 315–321 (2008).
Connolly, 2006	ACTIVE W	(3) ACTIVE Writing Group of the ACTIVE Investigators *et al.* Clopidogrel plus aspirin versus oral anticoagulation for atrial fibrillation in the Atrial fibrillation Clopidogrel Trial with Irbesartan for prevention of Vascular Events (ACTIVE W): a randomised controlled trial. *Lancet Lond. Engl.* **367**, 1903–1912 (2006).
Connolly, 2011	AVERROES	(4) Connolly, S. J. *et al.* Apixaban in patients with atrial fibrillation. *N. Engl. J. Med.* **364**, 806–817 (2011).
Connolly, 1991	CAFA	(5) Connolly, S. J. *et al.* Canadian Atrial Fibrillation Anticoagulation (CAFA) Study. *J. Am. Coll. Cardiol.* **18**, 349–355 (1991)
Connolly, 2009	RE-LY	(6) Connolly, S. J. *et al.* Dabigatran versus Warfarin in Patients with Atrial Fibrillation. *N. Engl. J. Med.* **361**, 1139–1151 (2009).
Diener, 1996	ESPS-2	(7) Diener, H. C. *et al.* European Stroke Prevention Study 2. Dipyridamole and acetylsalicylic acid in the secondary prevention of stroke11ESPS-2 Writing Committee. *J. Neurol. Sci.* **143**, 1–13 (1996).
Ezekowitz, 1992	SPINAF	(8) Ezekowitz, M. D. *et al.* Warfarin in the prevention of stroke associated with nonrheumatic atrial fibrillation. Veterans Affairs Stroke Prevention in Nonrheumatic Atrial Fibrillation Investigators. *N. Engl. J. Med.* **327**, 1406–1412 (1992).
Giugliano, 2013	ENGAGE AF-TIMI	(9) Giugliano, R. P. *et al.* Edoxaban versus warfarin in patients with atrial fibrillation. *N. Engl. J. Med.* **369**, 2093–2104 (2013).
Granger, 2011	ARISTOTLE	(10) Granger, C. B. *et al.* Apixaban versus warfarin in patients with atrial fibrillation. *N. Engl. J. Med.* **365**, 981–992 (2011).
Gullov, 1998	AFASAK II	(11) Gulløv, A. L. *et al.* Fixed minidose warfarin and aspirin alone and in combination vs adjusted-dose warfarin for stroke prevention in atrial fibrillation: Second Copenhagen Atrial Fibrillation, Aspirin, and Anticoagulation Study. *Arch. Intern. Med.* **158**, 1513–1521 (1998).
Halperin, 2005	SPORTIF V	(12) Giugliano, R. P. *et al.* Edoxaban versus warfarin in patients with atrial fibrillation. *N. Engl. J. Med.* **369**, 2093–2104 (2013).
Hellemons, 1999	PATAFa	(13) Hellemons, B. S. *et al.* Primary prevention of arterial thromboembolism in non-rheumatic atrial fibrillation in primary care: randomised controlled trial comparing two intensities of coumarin with aspirin. *BMJ* **319**, 958–964 (1999)
Hori, 2012	J-ROCKET AF	(14) Hori, M. *et al.* Rivaroxaban vs. warfarin in Japanese patients with atrial fibrillation – the J-ROCKET AF study –. *Circ. J. Off. J. Jpn. Circ. Soc.* **76**, 2104–2111 (2012).
Kistler, 1990	BAATAF	(15) Boston Area anticoagulation Trial for Atrial Fibrillation Investigators et al. The effect of low-dose warfarin on the risk of stroke in patients with nonrheumatic atrial fibrillation. N. Engl. J. Med. 323, 1505–1511 (1990).
Lip, 2009	NCT00684307	(16) Lip, G. Y. H. *et al.* Oral direct thrombin inhibitor AZD0837 for the prevention of stroke and systemic embolism in patients with non-valvular atrial fibrillation: a randomized dose-guiding, safety, and tolerability study of four doses of AZD0837 vs. vitamin K antagonists. *Eur. Heart J.* **30**, 2897–2907 (2009).
Mant, 2007	BAFTA	(17) Mant, J. *et al.* Warfarin versus aspirin for stroke prevention in an elderly community population with atrial fibrillation (the Birmingham Atrial Fibrillation Treatment of the Aged Study, BAFTA): a randomised controlled trial. *Lancet Lond. Engl.* **370**, 493–503 (2007).

Author, year	Study acronym	Full reference
McBride, 1991	SPAF I	(18) Stroke Prevention in Atrial Fibrillation Study. Final results. *Circulation* **84**, 527–539 (1991).
McBride, 1996	SPAF III	(19) Adjusted-dose warfarin versus low-intensity, fixed-dose warfarin plus aspirin for high-risk patients with atrial fibrillation: Stroke Prevention in Atrial Fibrillation III randomised clinical trial. *The Lancet* **348**, 633–638 (1996).
Morocutti, 1997	SIFA	(20) Morocutti, C. *et al.* Indobufen versus warfarin in the secondary prevention of major vascular events in nonrheumatic atrial fibrillation. SIFA (Studio Italiano Fibrillazione Atriale) Investigators. *Stroke* **28**, 1015–1021 (1997).
Olsson, 2003	SPORTIF III	(21) Olsson, S. B. & Executive Steering Committee of the SPORTIF III Investigators. Stroke prevention with the oral direct thrombin inhibitor ximelagatran compared with warfarin in patients with non-valvular atrial fibrillation (SPORTIF III): randomised controlled trial. *Lancet Lond. Engl.* **362**, 1691–1698 (2003).
Pérez-Gomez, 2004	NASPEAFa	(22) Pérez-Gómez, F. *et al.* Comparative effects of antiplatelet, anticoagulant, or combined therapy in patients with valvular and nonvalvular atrial fibrillation: A randomized multicenter study. *J. Am. Coll. Cardiol.* **44**, 1557–1566 (2004).
Pérez-Gomez, 2004	NASPEAFb	(22) Pérez-Gómez, F. *et al.* Comparative effects of antiplatelet, anticoagulant, or combined therapy in patients with valvular and nonvalvular atrial fibrillation: A randomized multicenter study. *J. Am. Coll. Cardiol.* **44**, 1557–1566 (2004).
Patel, 2011	ROCKET AF	(23) Patel, M. R. *et al.* Rivaroxaban versus Warfarin in Nonvalvular Atrial Fibrillation. *N. Engl. J. Med.* **365**, 883–891 (2011).
Pengo, 2010	/	(24) Pengo, V. *et al.* Lower versus standard intensity oral anticoagulant therapy (OAT) in elderly warfarin-experienced patients with non-valvular atrial fibrillation. *Thromb. Haemost.* **103**, 442–449 (2010).
Sato, 2006	JAST	(25) Sato, H. *et al.* Low-dose aspirin for prevention of stroke in low-risk patients with atrial fibrillation: Japan Atrial Fibrillation Stroke Trial. *Stroke* **37**, 447–451 (2006).
Connolly, 2013	EXPLORE-Xa	(26) Connolly, S. J. *et al.* Betrixaban compared with warfarin in patients with atrial fibrillation: results of a phase 2, randomized, dose-ranging study (Explore-Xa). *Eur. Heart J.* **34**, 1498–1505 (2013).

C.2 ACUTE VENOUS THROMBOEMBOLISM

Author, year	Study acronym	Full refference
Büller, 2012	CASSIOPEA	(27) Büller, H. R., Gallus, A. S., Pillion, G., Prins, M. H. & Raskob, G. E. Enoxaparin followed by once-weekly idrabiotaparinux versus enoxaparin plus warfarin for patients with acute symptomatic pulmonary embolism: a randomised, double-blind, double-dummy, non-inferiority trial. *The Lancet* **379**, 123–129 (2012).
Büller, 1997	COLOMBUS	(28) Columbus Investigators *et al.* Low-molecular-weight heparin in the treatment of patients with venous thromboembolism. *N. Engl. J. Med.* **337**, 657–662 (1997).
Büller, 2010	EINSTEIN DVT	(29) Oral Rivaroxaban for Symptomatic Venous Thromboembolism. *N. Engl. J. Med.* **363**, 2499–2510 (2010).
Büller, 2008	EINSTEIN DVT	(30) Buller, H. R. *et al.* A dose-ranging study evaluating once-daily oral administration of the factor Xa inhibitor rivaroxaban in the treatment of patients with acute symptomatic deep vein thrombosis: the Einstein-DVT Dose-Ranging Study. *Blood* **112**, 2242–2247 (2008).
Büller, 2012	EINSTEIN PE	(31) Oral Rivaroxaban for the Treatment of Symptomatic Pulmonary Embolism. *N. Engl. J. Med.* **366**, 1287–1297 (2012).
Büller, 2011	EQUINOX	(32) Equinox Investigators. Efficacy and safety of once weekly subcutaneous idrabiotaparinux in the treatment of patients with symptomatic deep venous thrombosis. *J. Thromb. Haemost. JTH* **9**, 92–99 (2011).

**Author, year**	**Study acronym**	**Full refference**
Büller, 2013	HOKUSAI VTE	(33) Hokusai-VTE Investigators *et al.* Edoxaban versus warfarin for the treatment of symptomatic venous thromboembolism. *N. Engl. J. Med.* **369**, 1406–1415 (2013).
Büller, 2004	MATISSE DVT	(34) Büller, H. R. *et al.* Fondaparinux or enoxaparin for the initial treatment of symptomatic deep venous thrombosis: a randomized trial. *Ann. Intern. Med.* **140**, 867–873 (2004).
Büller, 2003	MATISSE PE	(35) Büller, H. R. *et al.* Subcutaneous fondaparinux versus intravenous unfractionated heparin in the initial treatment of pulmonary embolism. *N. Engl. J. Med.* **349**, 1695–1702 (2003).
Büller, 2007	VAN GOGH DVT	(36) van Gogh Investigators *et al.* Idraparinux versus standard therapy for venous thromboembolic disease. *N. Engl. J. Med.* **357**, 1094–1104 (2007).
Büller, 2007	VAN GOGH PE	(36) van Gogh Investigators *et al.* Idraparinux versus standard therapy for venous thromboembolic disease. *N. Engl. J. Med.* **357**, 1094–1104 (2007).
Agnelli, 2013	AMPLIFY	(37) Agnelli, G. *et al.* Oral Apixaban for the Treatment of Acute Venous Thromboembolism. *N. Engl. J. Med.* **369**, 799–808 (2013).
Agnelli, 2013	AMPLIFY EXT	(38) Agnelli, G. *et al.* Apixaban for extended treatment of venous thromboembolism. *N. Engl. J. Med.* **368**, 699–708 (2013).
Brandjes, 1992	/	(39) Brandjes, D. P. M. *et al.* Acenocoumarol and Heparin Compared with Acenocoumarol Alone in the Initial Treatment of Proximal-Vein Thrombosis. *N. Engl. J. Med.* **327**, 1485–1489 (1992).
Hull, 1992	/	(40) Low-Molecular-Weight Heparin. *N. Engl. J. Med.* **327**, 817–818 (1992).
Kearon, 2006	FIDO	(41) Kearon, C. *et al.* Comparison of fixed-dose weight-adjusted unfractionated heparin and low-molecular-weight heparin for acute treatment of venous thromboembolism. *JAMA* **296**, 935–942 (2006).
Levine, 1996	/	(42) Levine, M. *et al.* A comparison of low-molecular-weight heparin administered primarily at home with unfractionated heparin administered in the hospital for proximal deep-vein thrombosis. *N. Engl. J. Med.* **334**, 677–681 (1996).
Merli, 2001	/	(43) Merli, G. *et al.* Subcutaneous enoxaparin once or twice daily compared with intravenous unfractionated heparin for treatment of venous thromboembolic disease. *Ann. Intern. Med.* **134**, 191–202 (2001).
Prandoni, 2004	GALILEI	(44) Prandoni, P., Carnovali, M., Marchiori, A. & Galilei Investigators. Subcutaneous adjusted-dose unfractionated heparin vs fixed-dose low-molecular-weight heparin in the initial treatment of venous thromboembolism. *Arch. Intern. Med.* **164**, 1077–1083 (2004).
Prandoni et al, 1992	/	(45) Anticoagulation-trials: PRANDONI. http://www.anticoagulant-trials.eu/studies-a-z/detail/study/prandoni.html
Schulman, 2009	RE-COVER	(46) Schulman, S. *et al.* Dabigatran versus Warfarin in the Treatment of Acute Venous Thromboembolism. *N. Engl. J. Med.* **361**, 2342–2352 (2009).
Schulman, 2013	RE-COVER II	(47) Schulman, S. *et al.* Treatment of acute venous thromboembolism with dabigatran or warfarin and pooled analysis. *Circulation* **129**, 764–772 (2014).
Schulman, 2013	RE-MEDY	(48) Schulman, S. *et al.* Extended use of dabigatran, warfarin, or placebo in venous thromboembolism. *N. Engl. J. Med.* **368**, 709–718 (2013).
Simonneau, 1997	THÉSÉE	(49) Simonneau, G. *et al.* A Comparison of Low-Molecular-Weight Heparin with Unfractionated Heparin for Acute Pulmonary Embolism. *N. Engl. J. Med.* **337**, 663–669 (1997).
Büller, 2007	VAN GOGH EXT	(50) van Gogh Investigators et al. Extended prophylaxis of venous thromboembolism with idraparinux. N. Engl. J. Med. 357, 1105–1112 (2007).
Eriksson, 2003	THRIVE I	(51) Eriksson, H. et al. A randomized, controlled, dose-guiding study of the oral direct thrombin inhibitor ximelagatran compared with standard therapy for the treatment of acute deep vein thrombosis: THRIVE I. J. Thromb. Haemost. 1, 41–47 (2003).

Author, year	Study acronym	Full refference
Fiessinger, 2005	THRIVE	(52) Fiessinger, J.-N. et al. Ximelagatran vs low-molecular-weight heparin and warfarin for the treatment of deep vein thrombosis: a randomized trial. JAMA 293, 681–689 (2005).

C.3 THROMBOPROPHYLAXIS IN PATIENTS HOSPITALIZED FOR MEDICAL CONDITIONS

Author, year	Study acronym	Full refference
Leizorovicz, 2004	PREVENT	(53) Leizorovicz, A. et al. Randomized, placebo-controlled trial of dalteparin for the prevention of venous thromboembolism in acutely ill medical patients. Circulation 110, 874–879 (2004).
Lederle, 2006	/	(54) Lederle, F. A. et al. The prophylaxis of medical patients for thromboembolism pilot study. Am. J. Med. 119, 54–59 (2006).
Samama, 1999	MEDENOX	(55) Samama, M. M. et al. A comparison of enoxaparin with placebo for the prevention of venous thromboembolism in acutely ill medical patients. Prophylaxis in Medical Patients with Enoxaparin Study Group. N. Engl. J. Med. 341, 793–800 (1999).
Fraisse, 2000	/	(56) Fraisse, F. et al. Nadroparin in the prevention of deep vein thrombosis in acute decompensated COPD. The Association of Non-University Affiliated Intensive Care Specialist Physicians of France. Am. J. Respir. Crit. Care Med. 161, 1109–1114 (2000).
Mahe, 2005	/	(57) Mahé, I., Bergmann, J. F., d'Azémar, P., Vaissie, J. J. & Caulin, C. Lack of effect of a low-molecular-weight heparin (nadroparin) on mortality in bedridden medical in-patients: a prospective randomised double-blind study. Eur. J. Clin. Pharmacol. 61, 347–351 (2005).
Diener, 2006	PROTECT	(58) Diener, H.-C. et al. Prophylaxis of thrombotic and embolic events in acute ischemic stroke with the low-molecular-weight heparin certoparin: results of the PROTECT Trial. Stroke 37, 139–144 (2006).
Riess, 2010	CERTIFY	(59) Riess, H. et al. A randomized, double-blind study of certoparin vs. unfractionated heparin to prevent venous thromboembolic events in acutely ill, non-surgical patients: CERTIFY Study. J. Thromb. Haemost. JTH 8, 1209–1215 (2010).
Schellong, 2010	CERTAIN	(60) Schellong, S. M. et al. An open-label comparison of the efficacy and safety of certoparin versus unfractionated heparin for the prevention of thromboembolic complications in acutely ill medical patients: CERTAIN. Expert Opin. Pharmacother. 11, 2953–2961 (2010).
Harenberg, 1990	/	(61) Harenberg, J. et al. Randomized controlled study of heparin and low molecular weight heparin for prevention of deep-vein thrombosis in medical patients. Thromb. Res. 59, 639–650 (1990).
Hillbom, 2002	/	(62) Hillbom, M. et al. Enoxaparin vs heparin for prevention of deep-vein thrombosis in acute ischaemic stroke: a randomized, double-blind study. Acta Neurol. Scand. 106, 84–92 (2002).
Sherman, 2007	PREVAIL	(63) Sherman, D. G. et al. The efficacy and safety of enoxaparin versus unfractionated heparin for the prevention of venous thromboembolism after acute ischaemic stroke (PREVAIL Study): an open-label randomised comparison. Lancet Lond. Engl. 369, 1347–1355 (2007).
Bergmann, 1996	EMSG	(64) Bergmann, J. F. & Neuhart, E. A multicenter randomized double-blind study of enoxaparin compared with unfractionated heparin in the prevention of venous thromboembolic disease in elderly in-patients bedridden for an acute medical illness. The Enoxaparin in Medicine Study Group. Thromb. Haemost. 76, 529–534 (1996).
Lechler, 1996	PRIME	(65) Lechler, E., Schramm, W. & Flosbach, C. W. The venous thrombotic risk in non-surgical patients: epidemiological data and efficacy/safety profile of a low-

Author, year	Study acronym	Full refference
		molecular-weight heparin (enoxaparin). The Prime Study Group. Haemostasis 26 Suppl 2, 49–56 (1996).
Kleber, 2003	PRINCE	(66) Kleber, F.-X. et al. Randomized comparison of enoxaparin with unfractionated heparin for the prevention of venous thromboembolism in medical patients with heart failure or severe respiratory disease. Am. Heart J. 145, 614–621 (2003).
Aquino, 1990	/	(67) TrialResults-center.org. Aquino et al., 1990. http://www.trialresultscenter.org/Aquino et al. trial-S15522 http://www.trialresultscenter.org/Aquino et al. trial-S15522 (2017).
Manciet, 1990	APTE	(68) Manciet G et al. A study of the efficacy and tolerance of Fraxiparine during long term prophylaxis of the elderly. In: Bounameaux H, Samama MM, Cate JW editor(s). Fraxiparine Second International Symposium. Recent Pharmacological and Clinical Data. New York: Schattauer. (1990).
Forette, 1995	/	(69) Forette, B. & Wolmark, Y. [Calcium nadroparin in the prevention of thromboembolic disease in elderly subjects. Study of tolerance]. Presse Medicale Paris Fr. 1983 24, 567–571 (1995).
Harenberg, 1996	HESIM	(70) Harenberg, J., Roebruck, P. & Heene, D. L. Subcutaneous low-molecular-weight heparin versus standard heparin and the prevention of thromboembolism in medical inpatients. The Heparin Study in Internal Medicine Group. Haemostasis 26, 127–139 (1996).
Gardlund, 1996	/	(71) Gärdlund, B. Randomised, controlled trial of low-dose heparin for prevention of fatal pulmonary embolism in patients with infectious diseases. The Heparin Prophylaxis Study Group. Lancet Lond. Engl. 347, 1357–1361 (1996).
Cohen, 2006	ARTEMIS	(72) Cohen, A. T. et al. Efficacy and safety of fondaparinux for the prevention of venous thromboembolism in older acute medical patients: randomised placebo controlled trial. BMJ 332, 325–329 (2006).
Goldhaber, 2011	ADOPT	(73) Goldhaber, S. Z. et al. Apixaban versus enoxaparin for thromboprophylaxis in medically ill patients. N. Engl. J. Med. 365, 2167–2177 (2011).
Cohen, 2013	MAGELLAN	(74) Cohen, A. T. et al. Rivaroxaban for Thromboprophylaxis in Acutely Ill Medical Patients. N. Engl. J. Med. 368, 513–523 (2013).
Cohen, 2016	APEX	(75) Cohen, A. T. et al. Extended Thromboprophylaxis with Betrixaban in Acutely Ill Medical Patients. N. Engl. J. Med. 375, 534–544 (2016).
Hull, 2010	EXCLAIM	(76) Hull, R. D. et al. Extended-duration venous thromboembolism prophylaxis in acutely ill medical patients with recently reduced mobility: a randomized trial. Ann. Intern. Med. 153, 8–18 (2010).
Spyropoulos, 2018	MARINER	(77) Spyropoulos, A. C. et al. Rivaroxaban for Thromboprophylaxis after Hospitalization for Medical Illness. N. Engl. J. Med. 379, 1118–1127 (2018).

C.4 MAJOR ORTHOPEDIC AND ABDOMINAL SURGERY

Author, year	Study acronym	Full refference
Levine, 1996	/	(78) Levine, M. N. *et al.* Ardeparin (low-molecular-weight heparin) vs graduated compression stockings for the prevention of venous thromboembolism. A randomized trial in patients undergoing knee surgery. *Arch. Intern. Med.* **156**, 851–856 (1996).
Leclerc, 1992	/	(79) Leclerc, J. R. *et al.* Prevention of deep vein thrombosis after major knee surgery--a randomized, double-blind trial comparing a low molecular weight heparin fragment (enoxaparin) to placebo. *Thromb. Haemost.* **67**, 417–423 (1992).

Author, year	Study acronym	Full refference
Colwell, 1994	/	(80) Colwell, C. W. *et al.* Use of enoxaparin, a low-molecular-weight heparin, and unfractionated heparin for the prevention of deep venous thrombosis after elective hip replacement. A clinical trial comparing efficacy and safety. Enoxaparin Clinical Trial Group. *J. Bone Joint Surg. Am.* **76**, 3–14 (1994).
Leyvraz A VALIDER, 1991	/	(81) Leyvraz, P. F. *et al.* Prevention of deep vein thrombosis after hip replacement: randomised comparison between unfractionated heparin and low molecular weight heparin. *BMJ* **303**, 543–548 (1991).
Haas, 2006	/	(82) Haas, S. *et al.* Prevention of major venous thromboembolism following total hip or knee replacement: a randomized comparison of low-molecular-weight heparin with unfractionated heparin (ECHOS Trial). *Int. Angiol. J. Int. Union Angiol.* **25**, 335–342 (2006).
Colwell, 1999	/	(83) Colwell, C. W. *et al.* Comparison of enoxaparin and warfarin for the prevention of venous thromboembolic disease after total hip arthroplasty. Evaluation during hospitalization and three months after discharge. *J. Bone Joint Surg. Am.* **81**, 932–940 (1999).
Fitzgerald, 2001	/	(84) Fitzgerald, R. H. *et al.* Prevention of venous thromboembolic disease following primary total knee arthroplasty. A randomized, multicenter, open-label, parallel-group comparison of enoxaparin and warfarin. *J. Bone Joint Surg. Am.* **83**, 900–906 (2001).
Leclerc, 1996	/	(85) Leclerc, J. R. *et al.* Prevention of venous thromboembolism after knee arthroplasty. A randomized, double-blind trial comparing enoxaparin with warfarin. *Ann. Intern. Med.* **124**, 619–626 (1996).
Lassen, 2009	ADVANCE-1	(86) Lassen, M. R. *et al.* Apixaban or enoxaparin for thromboprophylaxis after knee replacement. *N. Engl. J. Med.* **361**, 594–604 (2009).
Lassen, 2010	ADVANCE-2	(87) Lassen, M. R. *et al.* Apixaban versus enoxaparin for thromboprophylaxis after knee replacement (ADVANCE-2): a randomised double-blind trial. *Lancet Lond. Engl.* **375**, 807–815 (2010).
Lassen, 2010	ADVANCE-3	(88) Lassen, M. R. *et al.* Apixaban versus enoxaparin for thromboprophylaxis after hip replacement. *N. Engl. J. Med.* **363**, 2487–2498 (2010).
Eriksson, 2007	RE-MODEL	(89) Eriksson, B. I. *et al.* Oral dabigatran etexilate vs. subcutaneous enoxaparin for the prevention of venous thromboembolism after total knee replacement: the RE-MODEL randomized trial. *J. Thromb. Haemost. JTH* **5**, 2178–2185 (2007).
Eriksson, 2007	RE-NOVATE	(90) Eriksson, B. I. *et al.* Dabigatran etexilate versus enoxaparin for prevention of venous thromboembolism after total hip replacement: a randomised, double-blind, non-inferiority trial. *Lancet Lond. Engl.* **370**, 949–956 (2007).
Eriksson, 2010	RE-NOVATE-2	(91) Eriksson, B. I. *et al.* Oral dabigatran versus enoxaparin for thromboprophylaxis after primary total hip arthroplasty (RE-NOVATE II*). A randomised, double-blind, non-inferiority trial. *Thromb. Haemost.* **105**, 721–729 (2011).
Ginsberg, 2008	RE-MOBILIZE	(92) RE-MOBILIZE Writing Committee *et al.* Oral thrombin inhibitor dabigatran etexilate vs North American enoxaparin regimen for prevention of venous thromboembolism after knee arthroplasty surgery. *J. Arthroplasty* **24**, 1–9 (2009).
Eriksson, 2008	RECORD 1	(93) Eriksson, B. I. *et al.* Rivaroxaban versus enoxaparin for thromboprophylaxis after hip arthroplasty. *N. Engl. J. Med.* **358**, 2765–2775 (2008).

**Author, year**	**Study acronym**	**Full refference**
Lassen, 2008	RECORD 3	(94) Lassen, M. R. *et al.* Rivaroxaban versus enoxaparin for thromboprophylaxis after total knee arthroplasty. *N. Engl. J. Med.* **358**, 2776–2786 (2008).
Turpie, 2009	RECORD 4	(95) Turpie, A. G. G. *et al.* Rivaroxaban versus enoxaparin for thromboprophylaxis after total knee arthroplasty (RECORD4): a randomised trial. *Lancet Lond. Engl.* **373**, 1673–1680 (2009).
Bauer, 2001	PENTAMAKS	(96) Bauer, K. A., Eriksson, B. I., Lassen, M. R., Turpie, A. G. & Steering Committee of the Pentasaccharide in Major Knee Surgery Study. Fondaparinux compared with enoxaparin for the prevention of venous thromboembolism after elective major knee surgery. *N. Engl. J. Med.* **345**, 1305–1310 (2001).
Eriksson, 2001	PENTHIFRA	(97) Eriksson, B. I., Bauer, K. A., Lassen, M. R., Turpie, A. G. & Steering Committee of the Pentasaccharide in Hip-Fracture Surgery Study. Fondaparinux compared with enoxaparin for the prevention of venous thromboembolism after hip-fracture surgery. *N. Engl. J. Med.* **345**, 1298–1304 (2001).
Lassen, 2002	EPHESUS	(98) Lassen, M. R., Bauer, K. A., Eriksson, B. I., Turpie, A. G. G. & European Pentasaccharide Elective Surgery Study (EPHESUS) Steering Committee. Postoperative fondaparinux versus preoperative enoxaparin for prevention of venous thromboembolism in elective hip-replacement surgery: a randomised double-blind comparison. *Lancet Lond. Engl.* **359**, 1715–1720 (2002).
Turpie, 2002	PENTATHLON	(99) Turpie, A. G. G., Bauer, K. A., Eriksson, B. I., Lassen, M. R. & PENTATHALON 2000 Study Steering Committee. Postoperative fondaparinux versus postoperative enoxaparin for prevention of venous thromboembolism after elective hip-replacement surgery: a randomised double-blind trial. *Lancet Lond. Engl.* **359**, 1721–1726 (2002).
Heit, 2000	/	(100) Heit, J. A. *et al.* Ardeparin sodium for extended out-of-hospital prophylaxis against venous thromboembolism after total hip or knee replacement. A randomized, double-blind, placebo-controlled trial. *Ann. Intern. Med.* **132**, 853–861 (2000).
Comp, 2001	/	(101) Comp, P. C. *et al.* Prolonged enoxaparin therapy to prevent venous thromboembolism after primary hip or knee replacement. Enoxaparin Clinical Trial Group. *J. Bone Joint Surg. Am.* **83**, 336–345 (2001).
Eriksson, 2003	PENTHIFRA-Plus	(102) Eriksson, B. I., Lassen, M. R. & PENTasaccharide in HIp-FRActure Surgery Plus Investigators. Duration of prophylaxis against venous thromboembolism with fondaparinux after hip fracture surgery: a multicenter, randomized, placebo-controlled, double-blind study. *Arch. Intern. Med.* **163**, 1337–1342 (2003).
Kakkar, 2008	RECORD2	(103) Kakkar, A. K. *et al.* Extended duration rivaroxaban versus short-term enoxaparin for the prevention of venous thromboembolism after total hip arthroplasty: a double-blind, randomised controlled trial. *Lancet Lond. Engl.* **372**, 31–39 (2008).
Anderson, 2018	/	(104) Anderson, D. R. *et al.* Aspirin or Rivaroxaban for VTE Prophylaxis after Hip or Knee Arthroplasty. *N. Engl. J. Med.* **378**, 699–707 (2018).
Lassen, 2012	SAVE-HIP1	(105) Lassen, M. R. *et al.* Semuloparin for prevention of venous thromboembolism after major orthopedic surgery: results from three

Author, year	Study acronym	Full refference
		randomized clinical trials, SAVE-HIP1, SAVE-HIP2 and SAVE-KNEE. *J. Thromb. Haemost. JTH* **10**, 822–832 (2012).
Lassen, 2012	SAVE-HIP2	(105) Lassen, M. R. *et al.* Semuloparin for prevention of venous thromboembolism after major orthopedic surgery: results from three randomized clinical trials, SAVE-HIP1, SAVE-HIP2 and SAVE-KNEE. *J. Thromb. Haemost. JTH* **10**, 822–832 (2012).
Lassen, 2012	SAVE-KNEE	(105) Lassen, M. R. *et al.* Semuloparin for prevention of venous thromboembolism after major orthopedic surgery: results from three randomized clinical trials, SAVE-HIP1, SAVE-HIP2 and SAVE-KNEE. *J. Thromb. Haemost. JTH* **10**, 822–832 (2012).
Fisher, 2013	SAVE-HIP3	(106) Fisher, W. D. *et al.* Extended venous thromboembolism prophylaxis in patients undergoing hip fracture surgery - the SAVE-HIP3 study. *Bone Jt. J.* **95-B**, 459–466 (2013).
Ho, 1999	/	(107) Ho, Y. H. *et al.* Randomized, controlled trial of low molecular weight heparin vs. no deep vein thrombosis prophylaxis for major colon and rectal surgery in Asian patients. *Dis. Colon Rectum* **42**, 196–202; discussion 202-203 (1999).
Rasmussen, 2006	FAME	(108) Rasmussen, M. S. *et al.* Prolonged prophylaxis with dalteparin to prevent late thromboembolic complications in patients undergoing major abdominal surgery: a multicenter randomized open-label study. *J. Thromb. Haemost. JTH* **4**, 2384–2390 (2006).
Bergqvist, 2002	ENOXACAN II	(109) Bergqvist, D. *et al.* Duration of prophylaxis against venous thromboembolism with enoxaparin after surgery for cancer. *N. Engl. J. Med.* **346**, 975–980 (2002).
Kakkar, 2010	CANBESURE	(110) Kakkar, V. V., Balibrea, J. L., Martínez-González, J., Prandoni, P. & CANBESURE Study Group. Extended prophylaxis with bemiparin for the prevention of venous thromboembolism after abdominal or pelvic surgery for cancer: the CANBESURE randomized study. *J. Thromb. Haemost. JTH* **8**, 1223–1229 (2010).
Turpie, 2007	APOLLO	(111) Turpie, A. G. G. *et al.* Fondaparinux combined with intermittent pneumatic compression vs. intermittent pneumatic compression alone for prevention of venous thromboembolism after abdominal surgery: a randomized, double-blind comparison. *J. Thromb. Haemost. JTH* **5**, 1854–1861 (2007).
Agnelli, 2005	PEGASUS	(112) Agnelli, G. *et al.* Randomized clinical trial of postoperative fondaparinux versus perioperative dalteparin for prevention of venous thromboembolism in high-risk abdominal surgery. *Br. J. Surg.* **92**, 1212–1220 (2005).
Kakkar, 2013	SAVE-ABDO	(113) Kakkar, A. K. *et al.* Preoperative enoxaparin versus postoperative semuloparin thromboprophylaxis in major abdominal surgery: a randomized controlled trial. *Ann. Surg.* **259**, 1073–1079 (2014).

C.5 THROMBOPROPHYLAXIS IN CANCER PATIENTS

Author, year	Study acronym	Full refference
Haas, 2012	TOPIC-1	(114) Haas, S. K. *et al.* Low-molecular-weight heparin versus placebo for the prevention of venous thromboembolism in metastatic breast cancer or stage

		III/IV lung cancer. *Clin. Appl. Thromb. Off. J. Int. Acad. Clin. Appl. Thromb.* **18**, 159–165 (2012).
Haas, 2012	TOPIC-2	(114) Haas, S. K. *et al.* Low-molecular-weight heparin versus placebo for the prevention of venous thromboembolism in metastatic breast cancer or stage III/IV lung cancer. *Clin. Appl. Thromb. Off. J. Int. Acad. Clin. Appl. Thromb.* **18**, 159–165 (2012).
Perry, 2010	PRODIGE	(115) Perry, J. R. *et al.* PRODIGE: a randomized placebo-controlled trial of dalteparin low-molecular-weight heparin thromboprophylaxis in patients with newly diagnosed malignant glioma. *J. Thromb. Haemost. JTH* **8**, 1959–1965 (2010).
Maraveyas, 2012	FRAGEM	(116) Maraveyas, A. *et al.* Gemcitabine versus gemcitabine plus dalteparin thromboprophylaxis in pancreatic cancer. *Eur. J. Cancer* **48**, 1283–1292 (2012).
Klerk, 2005	MALT	(117) Klerk, C. P. W. *et al.* The Effect of Low Molecular Weight Heparin on Survival in Patients With Advanced Malignancy. *J. Clin. Oncol.* **23**, 2130–2135 (2005).
Agnelli, 2009	PROTECHT	(118) Agnelli, G. *et al.* Nadroparin for the prevention of thromboembolic events in ambulatory patients with metastatic or locally advanced solid cancer receiving chemotherapy: a randomised, placebo-controlled, double-blind study. *Lancet Oncol.* **10**, 943–949 (2009).
van Doormaal, 2011	INPACT	(119) van Doormaal, F. F. *et al.* Randomized trial of the effect of the low molecular weight heparin nadroparin on survival in patients with cancer. *J. Clin. Oncol. Off. J. Am. Soc. Clin. Oncol.* **29**, 2071–2076 (2011).
Agnelli, 2012	SAVE-ONCO	(120) Agnelli, G. *et al.* Semuloparin for Thromboprophylaxis in Patients Receiving Chemotherapy for Cancer. *N. Engl. J. Med.* **366**, 601–609 (2012).

Refferences

1. Secondary prevention in non-rheumatic atrial fibrillation after transient ischaemic attack or minor stroke. EAFT (European Atrial Fibrillation Trial) Study Group. Lancet Lond Engl. 1993 Nov 20;342(8882):1255–62.

2. Amadeus Investigators, Bousser MG, Bouthier J, Büller HR, Cohen AT, Crijns H, et al. Comparison of idraparinux with vitamin K antagonists for prevention of thromboembolism in patients with atrial fibrillation: a randomised, open-label, non-inferiority trial. Lancet Lond Engl. 2008 Jan 26;371(9609):315–21.

3. ACTIVE Writing Group of the ACTIVE Investigators, Connolly S, Pogue J, Hart R, Pfeffer M, Hohnloser S, et al. Clopidogrel plus aspirin versus oral anticoagulation for atrial fibrillation in the Atrial fibrillation Clopidogrel Trial with Irbesartan for prevention of Vascular Events (ACTIVE W): a randomised controlled trial. Lancet Lond Engl. 2006 Jun 10;367(9526):1903–12.

4. Connolly SJ, Eikelboom J, Joyner C, Diener H-C, Hart R, Golitsyn S, et al. Apixaban in patients with atrial fibrillation. N Engl J Med. 2011 Mar 3;364(9):806–17.

5. Connolly SJ, Laupacis A, Gent M, Roberts RS, Cairns JA, Joyner C. Canadian Atrial Fibrillation Anticoagulation (CAFA) Study. J Am Coll Cardiol. 1991 Aug;18(2):349–55.

6. Connolly SJ, Ezekowitz MD, Yusuf S, Eikelboom J, Oldgren J, Parekh A, et al. Dabigatran versus Warfarin in Patients with Atrial Fibrillation. N Engl J Med. 2009 Sep 17;361(12):1139–51.

7. Diener HC, Cunha L, Forbes C, Sivenius J, Smets P, Lowenthal A. European Stroke Prevention Study 2. Dipyridamole and acetylsalicylic acid in the secondary prevention of stroke11ESPS-2 Writing Committee. J Neurol Sci. 1996 Nov 1;143(1):1–13.

8. Ezekowitz MD, Bridgers SL, James KE, Carliner NH, Colling CL, Gornick CC, et al. Warfarin in the prevention of stroke associated with nonrheumatic atrial fibrillation. Veterans Affairs Stroke Prevention in Nonrheumatic Atrial Fibrillation Investigators. N Engl J Med. 1992 Nov 12;327(20):1406–12.

9. Giugliano RP, Ruff CT, Braunwald E, Murphy SA, Wiviott SD, Halperin JL, et al. Edoxaban versus warfarin in patients with atrial fibrillation. N Engl J Med. 2013 Nov 28;369(22):2093–104.

10. Granger CB, Alexander JH, McMurray JJV, Lopes RD, Hylek EM, Hanna M, et al. Apixaban versus warfarin in patients with atrial fibrillation. N Engl J Med. 2011 Sep 15;365(11):981–92.

11. Gulløv AL, Koefoed BG, Petersen P, Pedersen TS, Andersen ED, Godtfredsen J, et al. Fixed minidose warfarin and aspirin alone and in combination vs adjusted-dose warfarin for stroke prevention in atrial fibrillation: Second Copenhagen Atrial Fibrillation, Aspirin, and Anticoagulation Study. Arch Intern Med. 1998 Jul 27;158(14):1513–21.

12. Albers GW, Diener H-C, Frison L, Grind M, Nevinson M, Partridge S, et al. Ximelagatran vs warfarin for stroke prevention in patients with nonvalvular atrial fibrillation: a randomized trial. JAMA. 2005 Feb 9;293(6):690–8.

13. Hellemons BS, Langenberg M, Lodder J, Vermeer F, Schouten HJ, Lemmens T, et al. Primary prevention of arterial thromboembolism in non-rheumatic atrial fibrillation in primary care: randomised controlled trial comparing two intensities of coumarin with aspirin. BMJ. 1999 Oct 9;319(7215):958–64.

14. Hori M, Matsumoto M, Tanahashi N, Momomura S, Uchiyama S, Goto S, et al. Rivaroxaban vs. warfarin in Japanese patients with atrial fibrillation – the J-ROCKET AF study –. Circ J Off J Jpn Circ Soc. 2012;76(9):2104–11.

15. Boston Area Anticoagulation Trial for Atrial Fibrillation Investigators, Singer DE, Hughes RA, Gress DR, Sheehan MA, Oertel LB, et al. The effect of low-dose warfarin on the risk of stroke in patients with nonrheumatic atrial fibrillation. N Engl J Med. 1990 29;323(22):1505–11.

16. Lip GYH, Rasmussen LH, Olsson SB, Jensen EC, Persson AL, Eriksson U, et al. Oral direct thrombin inhibitor AZD0837 for the prevention of stroke and systemic embolism in patients with non-valvular atrial fibrillation: a randomized dose-guiding, safety, and tolerability study of four doses of AZD0837 vs. vitamin K antagonists. Eur Heart J. 2009 Dec;30(23):2897–907.

17. Mant J, Hobbs FDR, Fletcher K, Roalfe A, Fitzmaurice D, Lip GYH, et al. Warfarin versus aspirin for stroke prevention in an elderly community population with atrial fibrillation (the Birmingham Atrial Fibrillation Treatment of the Aged Study, BAFTA): a randomised controlled trial. Lancet Lond Engl. 2007 Aug 11;370(9586):493–503.

18. Stroke Prevention in Atrial Fibrillation Study. Final results. Circulation. 1991 Aug;84(2):527–39.

19. Adjusted-dose warfarin versus low-intensity, fixed-dose warfarin plus aspirin for high-risk patients with atrial fibrillation: Stroke Prevention in Atrial Fibrillation III randomised clinical trial. The Lancet. 1996 Sep 7;348(9028):633–8.

20. Morocutti C, Amabile G, Fattapposta F, Nicolosi A, Matteoli S, Trappolini M, et al. Indobufen versus warfarin in the secondary prevention of major vascular events in nonrheumatic atrial fibrillation. SIFA (Studio Italiano Fibrillazione Atriale) Investigators. Stroke. 1997 May;28(5):1015–21.

21. Olsson SB, Executive Steering Committee of the SPORTIF III Investigators. Stroke prevention with the oral direct thrombin inhibitor ximelagatran compared with warfarin in patients with non-valvular atrial fibrillation (SPORTIF III): randomised controlled trial. Lancet Lond Engl. 2003 Nov 22;362(9397):1691–8.

22. Pérez-Gómez F, Alegría E, Berjón J, Iriarte JA, Zumalde J, Salvador A, et al. Comparative effects of antiplatelet, anticoagulant, or combined therapy in patients with valvular and nonvalvular atrial fibrillation: A randomized multicenter study. J Am Coll Cardiol. 2004 Oct 19;44(8):1557–66.

23. Patel MR, Mahaffey KW, Garg J, Pan G, Singer DE, Hacke W, et al. Rivaroxaban versus Warfarin in Nonvalvular Atrial Fibrillation. N Engl J Med. 2011 Sep 8;365(10):883–91.

24. Pengo V, Cucchini U, Denas G, Davidson BL, Marzot F, Jose SP, et al. Lower versus standard intensity oral anticoagulant therapy (OAT) in elderly warfarin-experienced patients with non-valvular atrial fibrillation. Thromb Haemost. 2010 Feb;103(2):442–9.

25. Sato H, Ishikawa K, Kitabatake A, Ogawa S, Maruyama Y, Yokota Y, et al. Low-dose aspirin for prevention of stroke in low-risk patients with atrial fibrillation: Japan Atrial Fibrillation Stroke Trial. Stroke. 2006 Feb;37(2):447–51.

26. Connolly SJ, Eikelboom J, Dorian P, Hohnloser SH, Gretler DD, Sinha U, et al. Betrixaban compared with warfarin in patients with atrial fibrillation: results of a phase 2, randomized, dose-ranging study (Explore-Xa). Eur Heart J. 2013 May;34(20):1498–505.

27. Büller HR, Gallus AS, Pillion G, Prins MH, Raskob GE. Enoxaparin followed by once-weekly idrabiotaparinux versus enoxaparin plus warfarin for patients with acute symptomatic pulmonary embolism: a randomised, double-blind, double-dummy, non-inferiority trial. The Lancet. 2012 Jan 14;379(9811):123–9.

28. Columbus Investigators, Büller HR, Gent M, Gallus AS, Ginsberg J, Prins MH, et al. Low-molecular-weight heparin in the treatment of patients with venous thromboembolism. N Engl J Med. 1997 04;337(10):657–62.

29. Oral Rivaroxaban for Symptomatic Venous Thromboembolism. N Engl J Med. 2010 Dec 23;363(26):2499–510.

30. Buller HR, Lensing AWA, Prins MH, Agnelli G, Cohen A, Gallus AS, et al. A dose-ranging study evaluating once-daily oral administration of the factor Xa inhibitor rivaroxaban in the treatment of patients with acute symptomatic deep vein thrombosis: the Einstein-DVT Dose-Ranging Study. Blood. 2008 Sep 15;112(6):2242–7.

31. Oral Rivaroxaban for the Treatment of Symptomatic Pulmonary Embolism. N Engl J Med. 2012 Apr 5;366(14):1287–97.

32. Equinox Investigators. Efficacy and safety of once weekly subcutaneous idrabiotaparinux in the treatment of patients with symptomatic deep venous thrombosis. J Thromb Haemost JTH. 2011 Jan;9(1):92–9.

33. Hokusai-VTE Investigators, Büller HR, Décousus H, Grosso MA, Mercuri M, Middeldorp S, et al. Edoxaban versus warfarin for the treatment of symptomatic venous thromboembolism. N Engl J Med. 2013 Oct 10;369(15):1406–15.

34. Büller HR, Davidson BL, Decousus H, Gallus A, Gent M, Piovella F, et al. Fondaparinux or enoxaparin for the initial treatment of symptomatic deep venous thrombosis: a randomized trial. Ann Intern Med. 2004 Jun 1;140(11):867–73.

35. Büller HR, Davidson BL, Decousus H, Gallus A, Gent M, Piovella F, et al. Subcutaneous fondaparinux versus intravenous unfractionated heparin in the initial treatment of pulmonary embolism. N Engl J Med. 2003 Oct 30;349(18):1695–702.

36. van Gogh Investigators, Buller HR, Cohen AT, Davidson B, Decousus H, Gallus AS, et al. Idraparinux versus standard therapy for venous thromboembolic disease. N Engl J Med. 2007 Sep 13;357(11):1094–104.

37. Agnelli G, Buller HR, Cohen A, Curto M, Gallus AS, Johnson M, et al. Oral Apixaban for the Treatment of Acute Venous Thromboembolism. N Engl J Med. 2013 Aug 29;369(9):799–808.

38. Agnelli G, Buller HR, Cohen A, Curto M, Gallus AS, Johnson M, et al. Apixaban for extended treatment of venous thromboembolism. N Engl J Med. 2013 21;368(8):699–708.

39. Brandjes DPM, Heijboer H, Büller HR, de Rijk M, Jagt H, ten Cate JW. Acenocoumarol and Heparin Compared with Acenocoumarol Alone in the Initial Treatment of Proximal-Vein Thrombosis. N Engl J Med. 1992 Nov 19;327(21):1485–9.

40. Low-Molecular-Weight Heparin. N Engl J Med. 1992 Sep 10;327(11):817–8.

41. Kearon C, Ginsberg JS, Julian JA, Douketis J, Solymoss S, Ockelford P, et al. Comparison of fixed-dose weight-adjusted unfractionated heparin and low-molecular-weight heparin for acute treatment of venous thromboembolism. JAMA. 2006 Aug 23;296(8):935–42.

42. Levine M, Gent M, Hirsh J, Leclerc J, Anderson D, Weitz J, et al. A comparison of low-molecular-weight heparin administered primarily at home with unfractionated heparin administered in the hospital for proximal deep-vein thrombosis. N Engl J Med. 1996 Mar 14;334(11):677–81.

43. Merli G, Spiro TE, Olsson CG, Abildgaard U, Davidson BL, Eldor A, et al. Subcutaneous enoxaparin once or twice daily compared with intravenous unfractionated heparin for treatment of venous thromboembolic disease. Ann Intern Med. 2001 Feb 6;134(3):191–202.

44. Prandoni P, Carnovali M, Marchiori A, Galilei Investigators. Subcutaneous adjusted-dose unfractionated heparin vs fixed-dose low-molecular-weight heparin in the initial treatment of venous thromboembolism. Arch Intern Med. 2004 May 24;164(10):1077–83.

45. Anticoagulation-trials: PRANDONI [Internet]. [cited 2020 Apr 1]. Available from: http://www.anticoagulant-trials.eu/studies-a-z/detail/study/prandoni.html

46. Schulman S, Kearon C, Kakkar AK, Mismetti P, Schellong S, Eriksson H, et al. Dabigatran versus Warfarin in the Treatment of Acute Venous Thromboembolism. N Engl J Med. 2009 Dec 10;361(24):2342–52.

47. Schulman S, Kakkar AK, Goldhaber SZ, Schellong S, Eriksson H, Mismetti P, et al. Treatment of acute venous thromboembolism with dabigatran or warfarin and pooled analysis. Circulation. 2014 Feb 18;129(7):764–72.

48. Schulman S, Kearon C, Kakkar AK, Schellong S, Eriksson H, Baanstra D, et al. Extended use of dabigatran, warfarin, or placebo in venous thromboembolism. N Engl J Med. 2013 Feb 21;368(8):709–18.

49. Simonneau G, Sors H, Charbonnier B, Page Y, Laaban J-P, Azarian R, et al. A Comparison of Low-Molecular-Weight Heparin with Unfractionated Heparin for Acute Pulmonary Embolism. N Engl J Med. 1997 Sep 4;337(10):663–9.

50. van Gogh Investigators, Buller HR, Cohen AT, Davidson B, Decousus H, Gallus AS, et al. Extended prophylaxis of venous thromboembolism with idraparinux. N Engl J Med. 2007 Sep 13;357(11):1105–12.

51. Eriksson H, Wåhlander K, Gustafsson D, Welin L t, Frison L, Schulman S. A randomized, controlled, dose-guiding study of the oral direct thrombin inhibitor ximelagatran compared with standard therapy for the treatment of acute deep vein thrombosis: THRIVE I. J Thromb Haemost. 2003;1(1):41–7.

52. Fiessinger J-N, Huisman MV, Davidson BL, Bounameaux H, Francis CW, Eriksson H, et al. Ximelagatran vs low-molecular-weight heparin and warfarin for the treatment of deep vein thrombosis: a randomized trial. JAMA. 2005 Feb 9;293(6):681–9.

53. Leizorovicz A, Cohen AT, Turpie AGG, Olsson C-G, Vaitkus PT, Goldhaber SZ, et al. Randomized, placebo-controlled trial of dalteparin for the prevention of venous thromboembolism in acutely ill medical patients. Circulation. 2004 Aug 17;110(7):874–9.

54. Lederle FA, Sacks JM, Fiore L, Landefeld CS, Steinberg N, Peters RW, et al. The prophylaxis of medical patients for thromboembolism pilot study. Am J Med. 2006 Jan;119(1):54–9.

55. Samama MM, Cohen AT, Darmon JY, Desjardins L, Eldor A, Janbon C, et al. A comparison of enoxaparin with placebo for the prevention of venous thromboembolism in acutely ill medical patients. Prophylaxis in Medical Patients with Enoxaparin Study Group. N Engl J Med. 1999 Sep 9;341(11):793–800.

56. Fraisse F, Holzapfel L, Couland JM, Simonneau G, Bedock B, Feissel M, et al. Nadroparin in the prevention of deep vein thrombosis in acute decompensated COPD. The Association of Non-University Affiliated Intensive Care Specialist Physicians of France. Am J Respir Crit Care Med. 2000 Apr;161(4 Pt 1):1109–14.

57. Mahé I, Bergmann JF, d'Azémar P, Vaissie JJ, Caulin C. Lack of effect of a low-molecular-weight heparin (nadroparin) on mortality in bedridden medical in-patients: a prospective randomised double-blind study. Eur J Clin Pharmacol. 2005 Jul;61(5–6):347–51.

58. Diener H-C, Ringelstein EB, von Kummer R, Landgraf H, Koppenhagen K, Harenberg J, et al. Prophylaxis of thrombotic and embolic events in acute ischemic stroke with the low-molecular-weight heparin certoparin: results of the PROTECT Trial. Stroke. 2006 Jan;37(1):139–44.

59. Riess H, Haas S, Tebbe U, Gerlach H-E, Abletshauser C, Sieder C, et al. A randomized, double-blind study of certoparin vs. unfractionated heparin to prevent venous thromboembolic

events in acutely ill, non-surgical patients: CERTIFY Study. J Thromb Haemost JTH. 2010 Jun;8(6):1209–15.

60. Schellong SM, Haas S, Greinacher A, Schwanebeck U, Sieder C, Abletshauser C, et al. An open-label comparison of the efficacy and safety of certoparin versus unfractionated heparin for the prevention of thromboembolic complications in acutely ill medical patients: CERTAIN. Expert Opin Pharmacother. 2010 Dec;11(18):2953–61.

61. Harenberg J, Kallenbach B, Martin U, Dempfle CE, Zimmermann R, Kübler W, et al. Randomized controlled study of heparin and low molecular weight heparin for prevention of deep-vein thrombosis in medical patients. Thromb Res. 1990 Aug 1;59(3):639–50.

62. Hillbom M, Erilä T, Sotaniemi K, Tatlisumak T, Sarna S, Kaste M. Enoxaparin vs heparin for prevention of deep-vein thrombosis in acute ischaemic stroke: a randomized, double-blind study. Acta Neurol Scand. 2002 Aug;106(2):84–92.

63. Sherman DG, Albers GW, Bladin C, Fieschi C, Gabbai AA, Kase CS, et al. The efficacy and safety of enoxaparin versus unfractionated heparin for the prevention of venous thromboembolism after acute ischaemic stroke (PREVAIL Study): an open-label randomised comparison. Lancet Lond Engl. 2007 Apr 21;369(9570):1347–55.

64. Bergmann JF, Neuhart E. A multicenter randomized double-blind study of enoxaparin compared with unfractionated heparin in the prevention of venous thromboembolic disease in elderly in-patients bedridden for an acute medical illness. The Enoxaparin in Medicine Study Group. Thromb Haemost. 1996 Oct;76(4):529–34.

65. Lechler E, Schramm W, Flosbach CW. The venous thrombotic risk in non-surgical patients: epidemiological data and efficacy/safety profile of a low-molecular-weight heparin (enoxaparin). The Prime Study Group. Haemostasis. 1996;26 Suppl 2:49–56.

66. Kleber F-X, Witt C, Vogel G, Koppenhagen K, Schomaker U, Flosbach CW, et al. Randomized comparison of enoxaparin with unfractionated heparin for the prevention of venous thromboembolism in medical patients with heart failure or severe respiratory disease. Am Heart J. 2003 Apr;145(4):614–21.

67. TrialResults-center.org. Aquino et al., 1990 [Internet]. http://www.trialresultscenter.org/Aquino et al. trial-S15522. TrialResults-center.org; 2017 [cited 2020 Apr 2]. Available from: http://www.trialresultscenter.org/Aquino et al. trial-S15522

68. Manciet G et al. A study of the efficacy and tolerance of Fraxiparine during long term prophylaxis of the elderly. In: Bounameaux H, Samama MM, Cate JW editor(s). Fraxiparine Second International Symposium. Recent Pharmacological and Clinical Data. New York: Schattauer. 1990;

69. Forette B, Wolmark Y. [Calcium nadroparin in the prevention of thromboembolic disease in elderly subjects. Study of tolerance]. Presse Medicale Paris Fr 1983. 1995 Mar 25;24(12):567–71.

70. Harenberg J, Roebruck P, Heene DL. Subcutaneous low-molecular-weight heparin versus standard heparin and the prevention of thromboembolism in medical inpatients. The Heparin Study in Internal Medicine Group. Haemostasis. 1996 Jun;26(3):127–39.

71. Gärdlund B. Randomised, controlled trial of low-dose heparin for prevention of fatal pulmonary embolism in patients with infectious diseases. The Heparin Prophylaxis Study Group. Lancet Lond Engl. 1996 May 18;347(9012):1357–61.

72. Cohen AT, Davidson BL, Gallus AS, Lassen MR, Prins MH, Tomkowski W, et al. Efficacy and safety of fondaparinux for the prevention of venous thromboembolism in older acute medical patients: randomised placebo controlled trial. BMJ. 2006 Feb 11;332(7537):325–9.

73. Goldhaber SZ, Leizorovicz A, Kakkar AK, Haas SK, Merli G, Knabb RM, et al. Apixaban versus enoxaparin for thromboprophylaxis in medically ill patients. N Engl J Med. 2011 Dec 8;365(23):2167–77.

74. Cohen AT, Spiro TE, Büller HR, Haskell L, Hu D, Hull R, et al. Rivaroxaban for Thromboprophylaxis in Acutely Ill Medical Patients. N Engl J Med. 2013 Feb 7;368(6):513–23.

75. Cohen AT, Harrington RA, Goldhaber SZ, Hull RD, Wiens BL, Gold A, et al. Extended Thromboprophylaxis with Betrixaban in Acutely Ill Medical Patients. N Engl J Med. 2016 11;375(6):534–44.

76. Hull RD, Schellong SM, Tapson VF, Monreal M, Samama M-M, Nicol P, et al. Extended-duration venous thromboembolism prophylaxis in acutely ill medical patients with recently reduced mobility: a randomized trial. Ann Intern Med. 2010 Jul 6;153(1):8–18.

77. Spyropoulos AC, Ageno W, Albers GW, Elliott CG, Halperin JL, Hiatt WR, et al. Rivaroxaban for Thromboprophylaxis after Hospitalization for Medical Illness. N Engl J Med. 2018 Sep 20;379(12):1118–27.

78. Levine MN, Gent M, Hirsh J, Weitz J, Turpie AG, Powers P, et al. Ardeparin (low-molecular-weight heparin) vs graduated compression stockings for the prevention of venous thromboembolism. A randomized trial in patients undergoing knee surgery. Arch Intern Med. 1996 Apr 22;156(8):851–6.

79. Leclerc JR, Geerts WH, Desjardins L, Jobin F, Laroche F, Delorme F, et al. Prevention of deep vein thrombosis after major knee surgery--a randomized, double-blind trial comparing a low molecular weight heparin fragment (enoxaparin) to placebo. Thromb Haemost. 1992 Apr 2;67(4):417–23.

80. Colwell CW, Spiro TE, Trowbridge AA, Morris BA, Kwaan HC, Blaha JD, et al. Use of enoxaparin, a low-molecular-weight heparin, and unfractionated heparin for the prevention of deep venous thrombosis after elective hip replacement. A clinical trial comparing efficacy and safety. Enoxaparin Clinical Trial Group. J Bone Joint Surg Am. 1994 Jan;76(1):3–14.

81. Leyvraz PF, Bachmann F, Hoek J, Büller HR, Postel M, Samama M, et al. Prevention of deep vein thrombosis after hip replacement: randomised comparison between unfractionated heparin and low molecular weight heparin. BMJ. 1991 Sep 7;303(6802):543–8.

82. Haas S, Breyer HG, Bacher HP, Fareed J, Misselwitz F, Victor N, et al. Prevention of major venous thromboembolism following total hip or knee replacement: a randomized comparison of low-molecular-weight heparin with unfractionated heparin (ECHOS Trial). Int Angiol J Int Union Angiol. 2006 Dec;25(4):335–42.

83. Colwell CW, Collis DK, Paulson R, McCutchen JW, Bigler GT, Lutz S, et al. Comparison of enoxaparin and warfarin for the prevention of venous thromboembolic disease after total hip

arthroplasty. Evaluation during hospitalization and three months after discharge. J Bone Joint Surg Am. 1999 Jul;81(7):932–40.

84. Fitzgerald RH, Spiro TE, Trowbridge AA, Gardiner GA, Whitsett TL, O'Connell MB, et al. Prevention of venous thromboembolic disease following primary total knee arthroplasty. A randomized, multicenter, open-label, parallel-group comparison of enoxaparin and warfarin. J Bone Joint Surg Am. 2001 Jun;83(6):900–6.

85. Leclerc JR, Geerts WH, Desjardins L, Laflamme GH, L'Espérance B, Demers C, et al. Prevention of venous thromboembolism after knee arthroplasty. A randomized, double-blind trial comparing enoxaparin with warfarin. Ann Intern Med. 1996 Apr 1;124(7):619–26.

86. Lassen MR, Raskob GE, Gallus A, Pineo G, Chen D, Portman RJ. Apixaban or enoxaparin for thromboprophylaxis after knee replacement. N Engl J Med. 2009 Aug 6;361(6):594–604.

87. Lassen MR, Raskob GE, Gallus A, Pineo G, Chen D, Hornick P, et al. Apixaban versus enoxaparin for thromboprophylaxis after knee replacement (ADVANCE-2): a randomised double-blind trial. Lancet Lond Engl. 2010 Mar 6;375(9717):807–15.

88. Lassen MR, Gallus A, Raskob GE, Pineo G, Chen D, Ramirez LM, et al. Apixaban versus enoxaparin for thromboprophylaxis after hip replacement. N Engl J Med. 2010 Dec 23;363(26):2487–98.

89. Eriksson BI, Dahl OE, Rosencher N, Kurth AA, van Dijk CN, Frostick SP, et al. Oral dabigatran etexilate vs. subcutaneous enoxaparin for the prevention of venous thromboembolism after total knee replacement: the RE-MODEL randomized trial. J Thromb Haemost JTH. 2007 Nov;5(11):2178–85.

90. Eriksson BI, Dahl OE, Rosencher N, Kurth AA, van Dijk CN, Frostick SP, et al. Dabigatran etexilate versus enoxaparin for prevention of venous thromboembolism after total hip replacement: a randomised, double-blind, non-inferiority trial. Lancet Lond Engl. 2007 Sep 15;370(9591):949–56.

91. Eriksson BI, Dahl OE, Huo MH, Kurth AA, Hantel S, Hermansson K, et al. Oral dabigatran versus enoxaparin for thromboprophylaxis after primary total hip arthroplasty (RE-NOVATE II*). A randomised, double-blind, non-inferiority trial. Thromb Haemost. 2011 Apr;105(4):721–9.

92. RE-MOBILIZE Writing Committee, Ginsberg JS, Davidson BL, Comp PC, Francis CW, Friedman RJ, et al. Oral thrombin inhibitor dabigatran etexilate vs North American enoxaparin regimen for prevention of venous thromboembolism after knee arthroplasty surgery. J Arthroplasty. 2009 Jan;24(1):1–9.

93. Eriksson BI, Borris LC, Friedman RJ, Haas S, Huisman MV, Kakkar AK, et al. Rivaroxaban versus enoxaparin for thromboprophylaxis after hip arthroplasty. N Engl J Med. 2008 Jun 26;358(26):2765–75.

94. Lassen MR, Ageno W, Borris LC, Lieberman JR, Rosencher N, Bandel TJ, et al. Rivaroxaban versus enoxaparin for thromboprophylaxis after total knee arthroplasty. N Engl J Med. 2008 Jun 26;358(26):2776–86.

95. Turpie AGG, Lassen MR, Davidson BL, Bauer KA, Gent M, Kwong LM, et al. Rivaroxaban versus enoxaparin for thromboprophylaxis after total knee arthroplasty (RECORD4): a randomised trial. Lancet Lond Engl. 2009 May 16;373(9676):1673–80.

96. Bauer KA, Eriksson BI, Lassen MR, Turpie AG, Steering Committee of the Pentasaccharide in Major Knee Surgery Study. Fondaparinux compared with enoxaparin for the prevention of venous thromboembolism after elective major knee surgery. N Engl J Med. 2001 Nov 1;345(18):1305–10.

97. Eriksson BI, Bauer KA, Lassen MR, Turpie AG, Steering Committee of the Pentasaccharide in Hip-Fracture Surgery Study. Fondaparinux compared with enoxaparin for the prevention of venous thromboembolism after hip-fracture surgery. N Engl J Med. 2001 Nov 1;345(18):1298–304.

98. Lassen MR, Bauer KA, Eriksson BI, Turpie AGG, European Pentasaccharide Elective Surgery Study (EPHESUS) Steering Committee. Postoperative fondaparinux versus preoperative enoxaparin for prevention of venous thromboembolism in elective hip-replacement surgery: a randomised double-blind comparison. Lancet Lond Engl. 2002 May 18;359(9319):1715–20.

99. Turpie AGG, Bauer KA, Eriksson BI, Lassen MR, PENTATHALON 2000 Study Steering Committee. Postoperative fondaparinux versus postoperative enoxaparin for prevention of venous thromboembolism after elective hip-replacement surgery: a randomised double-blind trial. Lancet Lond Engl. 2002 May 18;359(9319):1721–6.

100. Heit JA, Elliott CG, Trowbridge AA, Morrey BF, Gent M, Hirsh J. Ardeparin sodium for extended out-of-hospital prophylaxis against venous thromboembolism after total hip or knee replacement. A randomized, double-blind, placebo-controlled trial. Ann Intern Med. 2000 Jun 6;132(11):853–61.

101. Comp PC, Spiro TE, Friedman RJ, Whitsett TL, Johnson GJ, Gardiner GA, et al. Prolonged enoxaparin therapy to prevent venous thromboembolism after primary hip or knee replacement. Enoxaparin Clinical Trial Group. J Bone Joint Surg Am. 2001 Mar;83(3):336–45.

102. Eriksson BI, Lassen MR, PENTasaccharide in HIp-FRActure Surgery Plus Investigators. Duration of prophylaxis against venous thromboembolism with fondaparinux after hip fracture surgery: a multicenter, randomized, placebo-controlled, double-blind study. Arch Intern Med. 2003 Jun 9;163(11):1337–42.

103. Kakkar AK, Brenner B, Dahl OE, Eriksson BI, Mouret P, Muntz J, et al. Extended duration rivaroxaban versus short-term enoxaparin for the prevention of venous thromboembolism after total hip arthroplasty: a double-blind, randomised controlled trial. Lancet Lond Engl. 2008 Jul 5;372(9632):31–9.

104. Anderson DR, Dunbar M, Murnaghan J, Kahn SR, Gross P, Forsythe M, et al. Aspirin or Rivaroxaban for VTE Prophylaxis after Hip or Knee Arthroplasty. N Engl J Med. 2018 Feb 22;378(8):699–707.

105. Lassen MR, Fisher W, Mouret P, Agnelli G, George D, Kakkar A, et al. Semuloparin for prevention of venous thromboembolism after major orthopedic surgery: results from three randomized clinical trials, SAVE-HIP1, SAVE-HIP2 and SAVE-KNEE. J Thromb Haemost JTH. 2012 May;10(5):822–32.

106. Fisher WD, Agnelli G, George DJ, Kakkar AK, Lassen MR, Mismetti P, et al. Extended venous thromboembolism prophylaxis in patients undergoing hip fracture surgery - the SAVE-HIP3 study. Bone Jt J. 2013 Apr;95-B(4):459–66.

107. Ho YH, Seow-Choen F, Leong A, Eu KW, Nyam D, Teoh MK. Randomized, controlled trial of low molecular weight heparin vs. no deep vein thrombosis prophylaxis for major colon and rectal surgery in Asian patients. Dis Colon Rectum. 1999 Feb;42(2):196–202; discussion 202-203.

108. Rasmussen MS, Jorgensen LN, Wille-Jørgensen P, Nielsen JD, Horn A, Mohn AC, et al. Prolonged prophylaxis with dalteparin to prevent late thromboembolic complications in patients undergoing major abdominal surgery: a multicenter randomized open-label study. J Thromb Haemost JTH. 2006 Nov;4(11):2384–90.

109. Bergqvist D, Agnelli G, Cohen AT, Eldor A, Nilsson PE, Le Moigne-Amrani A, et al. Duration of prophylaxis against venous thromboembolism with enoxaparin after surgery for cancer. N Engl J Med. 2002 Mar 28;346(13):975–80.

110. Kakkar VV, Balibrea JL, Martínez-González J, Prandoni P, CANBESURE Study Group. Extended prophylaxis with bemiparin for the prevention of venous thromboembolism after abdominal or pelvic surgery for cancer: the CANBESURE randomized study. J Thromb Haemost JTH. 2010 Jun;8(6):1223–9.

111. Turpie AGG, Bauer KA, Caprini JA, Comp PC, Gent M, Muntz JE, et al. Fondaparinux combined with intermittent pneumatic compression vs. intermittent pneumatic compression alone for prevention of venous thromboembolism after abdominal surgery: a randomized, double-blind comparison. J Thromb Haemost JTH. 2007 Sep;5(9):1854–61.

112. Agnelli G, Bergqvist D, Cohen AT, Gallus AS, Gent M, PEGASUS investigators. Randomized clinical trial of postoperative fondaparinux versus perioperative dalteparin for prevention of venous thromboembolism in high-risk abdominal surgery. Br J Surg. 2005 Oct;92(10):1212–20.

113. Kakkar AK, Agnelli G, Fisher W, George D, Lassen MR, Mismetti P, et al. Preoperative enoxaparin versus postoperative semuloparin thromboprophylaxis in major abdominal surgery: a randomized controlled trial. Ann Surg. 2014 Jun;259(6):1073–9.

114. Haas SK, Freund M, Heigener D, Heilmann L, Kemkes-Matthes B, von Tempelhoff G-F, et al. Low-molecular-weight heparin versus placebo for the prevention of venous thromboembolism in metastatic breast cancer or stage III/IV lung cancer. Clin Appl Thromb Off J Int Acad Clin Appl Thromb. 2012 Apr;18(2):159–65.

115. Perry JR, Julian JA, Laperriere NJ, Geerts W, Agnelli G, Rogers LR, et al. PRODIGE: a randomized placebo-controlled trial of dalteparin low-molecular-weight heparin thromboprophylaxis in patients with newly diagnosed malignant glioma. J Thromb Haemost JTH. 2010 Sep;8(9):1959–65.

116. Maraveyas A, Waters J, Roy R, Fyfe D, Propper D, Lofts F, et al. Gemcitabine versus gemcitabine plus dalteparin thromboprophylaxis in pancreatic cancer. Eur J Cancer. 2012 Jun 1;48(9):1283–92.

117. Klerk CPW, Smorenburg SM, Otten H-M, Lensing AWA, Prins MH, Piovella F, et al. The Effect of Low Molecular Weight Heparin on Survival in Patients With Advanced Malignancy. J Clin Oncol. 2005 Apr 1;23(10):2130–5.

118. Agnelli G, Gussoni G, Bianchini C, Verso M, Mandalà M, Cavanna L, et al. Nadroparin for the prevention of thromboembolic events in ambulatory patients with metastatic or locally advanced solid cancer receiving chemotherapy: a randomised, placebo-controlled, double-blind study. Lancet Oncol. 2009 Oct;10(10):943–9.

119. van Doormaal FF, Di Nisio M, Otten H-M, Richel DJ, Prins M, Buller HR. Randomized trial of the effect of the low molecular weight heparin nadroparin on survival in patients with cancer. J Clin Oncol Off J Am Soc Clin Oncol. 2011 May 20;29(15):2071–6.

120. Agnelli G, George DJ, Kakkar AK, Fisher W, Lassen MR, Mismetti P, et al. Semuloparin for Thromboprophylaxis in Patients Receiving Chemotherapy for Cancer. N Engl J Med. 2012 Feb 16;366(7):601–9.
