# Supplementary material for: Meta-regression of randomized control trials with antithrombotics: weak correlation between net clinical benefit and all cause-mortality
Source: Sci Rep. 2021 Jul 19;11:14728. doi: 10.1038/s41598-021-94160-1 (PMC8290002; doi:10.1038/s41598-021-94160-1)
Supplement: Supplementary file 4 — Supplementary Information 4. [file 41598_2021_94160_MOESM4_ESM.pdf]

HIGHLIGHTS

- The net clinical benefit (NCB) is the sum of major bleeding and thrombotic events and has been used as the main outcome in phase III trials testing direct oral anticoagulants and may be viewed as a surrogate for all-cause mortality.
- A weak correlation between NCB and all cause-mortality was found in non-valvular atrial fibrillation (NVAF) and acute venous thromboembolism (VTE) patients. Additionally, no correlation was observed in prevention studies that included medical, surgical, and other prevention in cancer patients.
- NCB should not be considered as a validated surrogate outcome of all-cause mortality in NVAF, acute VTE, and VTE prevention trials.
- Major bleeding and thrombotic events caused only a small percentage of death in this field; also, the clinical impact of these events is not similar and has not the same weight and incidence according to the subset of cardiovascular diseases.
