# Supplementary material for: Meta-regression of randomized control trials with antithrombotics: weak correlation between net clinical benefit and all cause-mortality
Source: Sci Rep. 2021 Jul 19;11:14728. doi: 10.1038/s41598-021-94160-1 (PMC8290002; doi:10.1038/s41598-021-94160-1)
Supplement: Supplementary file 5 — Supplementary Information 5. [file 41598_2021_94160_MOESM5_ESM.pdf]

Search strategy

Pubmed:

(((anticoagulants[MeSH Terms]) AND (vitamin k antagonist[MeSH Terms])) OR (warfarin[MeSH Terms])) OR (semuloparin[MeSH Terms])) OR (fluindione[MeSH Terms])) OR (heparin[MeSH Terms])) OR (UFH[MeSH Terms])) OR (lmwh[MeSH Terms])) OR (aapixaban[MeSH Terms])) OR (betrixaban[MeSH Terms])) OR (dabigatran[MeSH Terms])) OR (rivaroxaban[MeSH Terms])) OR (dalteparin[MeSH Terms])) OR (edoxaban[MeSH Terms])) OR (AZD0837[MeSH Terms])) OR (acenocoumarol, idrabiotaparinux[MeSH Terms])) OR (fondaparinux[MeSH Terms])) OR (aspirin[MeSH Terms])) OR (dipyridamole[MeSH Terms])) OR (nadroparin[MeSH Terms])) OR (ticlopidine[MeSH Terms])) OR (ximelagatran[MeSH Terms])) OR (enoxaparin[MeSH Terms])) OR (reviparin[MeSH Terms])) OR (bemiparin[MeSH Terms])) OR (tinzaparin[MeSH Terms])) OR (certoparin[MeSH Terms])) OR (fraxiparin[MeSH Terms])) OR (adreparin[MeSH Terms])) AND (major bleeding[MeSH Terms])) OR (intracranial bleeding[MeSH Terms])) OR (gastrointestinal bleeding[MeSH Terms])) AND (thrombosis[MeSH Terms])) OR (venous thromboembolism[MeSH Terms])) OR (deep vein thrombosis[MeSH Terms])) OR (pulmonary embolism[MeSH Terms])) OR (atrial fibrillation[MeSH Terms])) OR (prophylaxis surgery[MeSH Terms])) OR (orthopedic surgery[MeSH Terms])) OR (major abdominal surgery[MeSH Terms])) OR (hip fractures[MeSH Terms])) OR (hip/knee surgery[MeSH Terms])) OR (hip/knee replacement[MeSH Terms])) OR (hip/knee arthroplasty[MeSH Terms])) OR (cancer[MeSH Terms])) OR (acute treatment[MeSH Terms])) AND (mortality[MeSH Terms])) OR (all-cause mortality[MeSH Terms])) AND (randomized controlled trial[MeSH Terms])) AND (net clinical benefit[MeSH Terms])
